# Remembering or monitoring? Contextual fit of simulated AI memory cues and consumer trust

**DOI:** 10.3389/fpsyg.2026.1934857

**Published:** 2026-09-10

**Authors:** Runhua Huang, Yanzhi Huang, Huichao Guo

**Affiliations:** 1 Business Technology Innovation and Development Research Center (Hangzhou), Zhejiang Business College, Hangzhou, Zhejiang, China; 2 School of Management and Economics, The Chinese University of Hong Kong, Shenzhen, Guangdong, China; 3 School of Safety Science and Emergency Management, Wuhan University of Technology, Wuhan, Hubei, China

**Keywords:** AI memory, boundary regulation, consumer trust, contextual integrity, personalization, privacy

## Abstract

AI assistants increasingly refer to information they ostensibly retain from earlier sessions. A remembered preference can signal attentiveness, whereas a reference to stress or late-night behavior can feel intrusive. Existing accounts of personalization and privacy do not fully explain why these cues elicit opposing reactions. We treat *Memory Boundary Fit* (MBF) as a preliminary interaction-specific operationalization of whether remembered information appears to fall within a consumer's contextual boundary for the current exchange. Across six controlled, LLM-delivered chat experiments (*N* = 2, 140), we compared three complete simulated message packages that differed in whether and how the assistant referenced remembered information: a relevant-memory cue, an intrusive-memory cue, and no memory reference. The assistant's memory references were fictitious claims rather than genuine retrieval of previously disclosed information. The principal and most consistent finding was that relevant-memory cue packages produced more favorable trust-related outcomes than intrusive-memory cue packages; comparisons with no memory varied across studies. Because the packages also differed in topical relevance, sensitivity, perceived usefulness, information source, and implied surveillance, the studies cannot isolate contextual fit, or any other single cue attribute, as the causal feature, and they do not establish MBF as a distinct psychological mechanism. Study 2 yielded same-session indirect associations consistent with the proposed MBF account. Across Study 2 and a supplemental validation sample, MBF explained additional variance in trust beyond that accounted for by the adjacent appraisals measured alongside it. However, all of these measures were self-reported in the same session. Study 3 found a larger intrusive-memory penalty in the complete sleep-health scenario than in the apparel scenario, but this is an exploratory scenario comparison rather than a test of information sensitivity. In Study 4, the specific editable dashboard tested increased average trust but did not selectively reduce the intrusive-memory penalty. Studies 5 and 6 extended the pattern to data-sharing and relational engagement, as well as to loyalty and opt-in intentions, but not to transactional engagement, which remained unaffected. These findings motivate contextual appropriateness as a candidate design consideration to be tested in real persistent-memory systems.

## 1 Introduction

Consider two replies to a user's request for headphone recommendations. The first says, “Since you told me you prefer a snug fit for running, here are three options.” The second says, “I noticed you've been browsing late at night and seem stressed lately. Maybe something calming would help?” Both replies use stored information, but the first is likely to feel helpful while the second may feel intrusive. The difference lies in whether the remembered information belongs in the exchange, rather than in how much the system remembers or how accurate it is.

AI assistants in shopping, travel, and health applications can already retain a user's budget, seating preference, recurring complaint, or mood cue across sessions. This capability can reduce repetition by allowing the system to reuse information the user has already provided. It also makes the reappearance of stored information part of the user's personalized experience.

Persistent memory makes stored data visible in the conversation. With conventional personalization, users may receive a tailored recommendation without seeing the data that produced it. A memory-enabled assistant, by contrast, can explicitly mention what it remembers, and the reference itself becomes a social cue. The same fact may signal attentiveness in one exchange and monitoring in another. This distinction aligns with evidence that willingness to share or reuse information depends heavily on the context in which it appears, not simply on its sensitivity (John et al., 2011). It also aligns with a recent study showing that data-driven personalization can elicit creepiness when consumers interpret it as intrusive or opaque surveillance (Petrova et al., 2026).

Consumer research often frames this tension as the personalization-privacy paradox: people value tailored experiences yet often resist the data practices needed to deliver them (Awad and Krishnan, 2006; Xu et al., 2011; Norberg et al., 2007). Privacy calculus models disclosure as a tradeoff between expected benefits and costs (Dinev and Hart, 2006), while procedural-fairness research shows that trust in an organization's information practices can offset some perceived costs (Culnan and Armstrong, 1999). These frameworks capture general evaluations of personalization and privacy, but visible AI memory raises a more specific question. A consumer may welcome one remembered detail and reject another in the same conversation. In that moment, the issue is not data collection in general but why the assistant knows a particular fact and whether it should mention it here. Privacy choices vary with framing, defaults, and immediate context (Acquisti et al., 2015; Adjerid et al., 2019). People also disclose the same information in one interactional frame and withhold it in another, even from an anonymous counterpart (John et al., 2011). A fixed disposition toward privacy concern does not readily explain either pattern.

The theory of contextual integrity locates privacy violations in information flows that breach contextual norms (Nissenbaum, 2010). On this account, a remembered detail is neither inherently private nor public. Its appropriateness depends on the people involved, the setting, and the purpose of the exchange. For AI-mediated consumer interactions, however, the theory does not, on its own, specify an interaction-level consumer measure. Such a measure is compatible with economic accounts of privacy tradeoffs (Acquisti et al., 2016) and with marketing research on data privacy and information control (Martin and Murphy, 2017). Both traditions treat privacy judgments as responsive to immediate circumstances.

Several adjacent traditions describe closely related reactions and therefore constrain what a new measure can claim. Communication privacy management treats disclosed information as jointly owned and governed by negotiated rules, so that a recipient who uses it outside those rules produces boundary turbulence (Petronio, 2002, 2013). Expectancy-violation accounts hold that a departure from what an interaction partner is expected to do is appraised positively or negatively depending on how the violation is interpreted (Burgoon, 1993). Research on advertising intrusiveness locates the harm in interruption and forced exposure (Edwards et al., 2002), whereas work on creepiness emphasizes practices that are lawful yet unsettling because social norms have not yet settled around them (Tene and Polonetsky, 2014; Petrova et al., 2026). These accounts converge on the premise that context governs the acceptability of an information flow. MBF is intended as a preliminary, compact interaction-level measure of that judgment for the AI-memory setting rather than as an established mechanism or a rival theory. Supplementary note S16 systematically compares MBF with each of these neighbors, identifying for each construct the specific judgment it targets, the condition under which it should diverge from MBF, and the measurement that would adjudicate between them. Compared with the appraisals measured alongside MBF in the present data, MBF showed incremental validity in its association with trust (Supplementary note S15). Because perceived usefulness, intrusiveness, creepiness, information relevance, and perceived norm violation were not measured, MBF's empirical separation from these appraisals remains an open question, and we return to it in the Construct boundaries section.

We refer to this situated judgment as *Memory Boundary Fit* (MBF). MBF is a preliminary, interaction-specific operationalization of whether remembered information is relevant, appropriate, and acceptable in the current exchange. General privacy concern is a relatively stable disposition toward data collection and use (Smith et al., 1996; Malhotra et al., 2004). By contrast, MBF concerns a particular use of remembered content. We conceptually distinguish MBF from task usefulness, which asks whether that content helps the immediate decision. A remembered detail may be useful yet seem out of place, or unobjectionable yet add little to the interaction. This conceptual distinction was not tested directly because perceived usefulness was not measured. MBF therefore provides a candidate framework for assessing whether remembered content belongs in a particular exchange.

MBF evaluates remembered content, whereas trust concerns beliefs about a system's competence, integrity, and benevolence under uncertainty (McKnight et al., 2002; Gefen et al., 2003; Pavlou, 2003). An assistant may accurately anticipate a user's needs and therefore appear competent. The same assistant may still lose integrity-based trust by surfacing information the user did not expect to see in the exchange. We therefore examine MBF as a candidate appraisal that determines whether a memory cue is perceived as attentiveness or surveillance.

Research on anthropomorphism and algorithmic skepticism suggests competing reactions to memory. Humanlike cues can make an automated agent seem warmer and more socially engaging (Blut et al., 2021), and remembering a previous exchange may reinforce that sense of continuity. Yet consumers often resist AI in decisions they regard as subjective, personal, or high stakes (Longoni et al., 2019; Castelo et al., 2019), although reactions vary by task and can include algorithm appreciation (Glikson and Woolley, 2020). A reference to stored information can therefore signal warmth and competence while reminding the user that the system captures data. This makes visible the dual role of AI as a social interlocutor and a tool for data collection (Puntoni et al., 2021).

Studies of recommendation agents show that personalization and familiarity can build trust by making an agent more useful and understandable (Komiak and Benbasat, 2006, 2008; Wang and Benbasat, 2005). Conversational framing can also substitute for trust during onboarding (Hildebrand and Bergner, 2021), even though users often struggle to determine how much weight to give a recommendation (Yeomans et al., 2019). Such familiarity has limits, as trust may decline when an agent knows or uses more information than the exchange requires. Human-computer interaction research also shows that users have expectations about what an agent should remember and how they should inspect, update, or correct its memory (Jones et al., 2025; Zhang et al., 2025). Long-term memory architectures for large language model agents provide useful capabilities but also introduce risks (Mekioussa Malki, 2026). This literature leaves open a consumer-behavior account linking memory design to trust, purchasing, data sharing, and loyalty intentions. The proposed MBF framework addresses this gap. AI memory also belongs to a wider class of connected consumer objects that retain data across uses (Hoffman and Novak, 2018). For these objects, concerns about personalization can involve consumer autonomy over a data trail as well as privacy narrowly defined (André et al., 2018).

The six studies follow a three-stage cumulative design. Studies 1 and 2 establish the memory-cue pattern, with Study 2 examining same-session indirect associations consistent with the proposed MBF account. Study 3 compares two complete scenarios, and Study 4 tests whether a specific editable dashboard selectively attenuates the intrusive-memory penalty. Studies 5 and 6 extend the analysis to data-sharing and relational engagement, privacy trust, and downstream intentions. Because participants had no prior interaction history with the assistant and the remembered details were fictitious, the experiments test consumer responses to simulated claims of memory rather than to genuine retrieval of previously disclosed information; we return to this boundary throughout.

The research makes three deliberately bounded contributions. First, it connects simulated memory-cue packages with consumer trust and engagement, extending work focused mainly on usability and privacy expectations (Jones et al., 2025; Zhang et al., 2025; Mekioussa Malki, 2026). Second, it applies an interaction-specific contextual account to the personalization-privacy tension by asking whether remembered content appears to belong in the current exchange (Awad and Krishnan, 2006; Xu et al., 2011). Third, it offers MBF as a preliminary consumer-level operationalization informed by contextual integrity (Nissenbaum, 2010). This measurement proposal is linked to commercially relevant outcomes, not evidence that a distinct psychological mechanism has been established.

### 1.1 Hypotheses

The studies address successive parts of the MBF account in three stages. They first test the memory-cue pattern and an indirect-association account, then compare complete scenarios with a single dashboard, and finally compare engagement and downstream intention outcomes. Relevant memory should increase trust by signaling competence and reducing effort. Intrusive memory should reduce trust because it may be interpreted as crossing a contextual boundary. No memory provides no personalization benefit but also avoids that potential boundary violation, so we expected it to fall between the other conditions. The relevant-vs.-intrusive contrast follows from these predictions and provides their clearest combined comparison.

**H1:** Relevant AI memory increases trust and purchase intention relative to no memory, whereas intrusive AI memory decreases both.

**H2:** Memory type is indirectly associated with trust through measured MBF. Because MBF and trust were measured in the same session, this hypothesis concerns a statistical indirect association rather than causal mediation.

Because consumers in high-sensitivity domains expect narrower information flows and stronger protection, we expected the harm of intrusive memory to be amplified rather than constant across product categories.

**H3:** The negative effect of intrusive memory on trust is stronger in high-sensitivity product domains than in low-sensitivity domains. Study 3 was motivated by this a priori expectation, but because its two complete scenarios varied across several attributes simultaneously, it offers only an exploratory scenario comparison and does not directly test H3.

If consumers can inspect, edit, or delete stored memories, memory use may be reinterpreted as procedurally fair and governable rather than imposed.

**H4:** An editable memory dashboard increases MBF and reduces the negative effect of intrusive memory on trust. As with H3, we retain the original a priori wording. Study 4 operationalized this expectation with a single inspect-edit-delete dashboard in one presentation and at one timing, and it included no perceived-control manipulation check, so its results are limited to that implementation rather than to editable control in general.

Data-sharing engagement exposes consumers to continued information vulnerability and should therefore depend strongly on trust in privacy practices. Perceived intrusiveness and privacy concerns can erode attitudes toward continued information use (Bano et al., 2022). Transactional engagement (e.g., purchase intention) often depends more on product utility and may be less sensitive to memory boundary violations.

**H5:** MBF has a stronger relationship with data-sharing engagement than with transactional engagement.

We expected this immediate relational evaluation to be associated with longer-run relationship outcomes via privacy trust.

**H6:** Memory type is associated with loyalty and opt-in intentions via serial indirect associations involving MBF and privacy trust.

## 2 Materials and methods

### 2.1 Participants and procedure

All six studies recruited students and local adult residents through Zhejiang Business College course-participation channels and on-campus community recruitment events. Students received course credit, and local residents received small gifts. Including both groups was intended to broaden the sample beyond students alone. Eligible participants were at least 18 years old, fluent in Chinese, and able to complete the scenario task. All materials, assistant messages, and questionnaire items were presented in Simplified Chinese. Data were collected between March and June 2026. At the beginning of each session, the experiment program assigned participants at the individual level using balanced randomized blocks; experimenters neither selected nor knew the assignments. Participants gave electronic informed consent before seeing the experimental material. Participants were told they were testing a prototype AI shopping assistant; the instructions made no claim about whether the assistant had access to previously stored personal data, and suspicion about the memory references was not assessed. The assistant's memory references were simulated, and participants had no prior interaction history with it. A debriefing screen therefore explained that the remembered details were fictitious and did not reflect stored personal data. The Institutional Review Board of Zhejiang Business College approved the protocol (Protocol No. ZJBCSCCT-2026-EXP007; March 1, 2026).

A total of 2,302 individuals were screened across the six studies. Screening applied four exclusion criteria: failed attention or comprehension checks; data-entry errors (incomplete, malformed, or duplicated submissions); insufficient completion time (less than one-third of the study median); and erroneous content (stimulus-delivery compliance failures or off-task free-text input). These criteria removed 162 responses (7.0%), leaving an analytic sample of *N* = 2, 140. Study-level samples were *n* = 264, *n* = 289, *n* = 410, *n* = 516, *n* = 318, and *n* = 343 for Studies 1–6, respectively. Supplementary Table S1 reports the screened, excluded, and retained counts for each study, along with the number of exclusions attributable to each of the four reasons. Because raw screening logs are stored separately from the de-identified analysis files under the IRB protocol, we report exclusions by study and by reason rather than by condition. The retained condition cells were nearly balanced in every study and show no obvious gross depletion of any single condition, but condition-specific exclusion rates cannot be tested from the retained files. Retained cell sizes were 91/88/85 (no-memory/relevant/intrusive) in Study 1, 96/99/94 in Study 2, 67–70 per cell across the six design cells in Study 3, 84–88 per cell in Study 4, 108/106/104 in Study 5, and 116/115/112 in Study 6. Because participation was anonymous and no directly identifying information was retained, we cannot verify whether any individual took part in more than one study. The pooled sample comprised 71.45% students and 28.55% local adult residents. Participants had a mean age of 26.18 years (*SD* = 10.61); 58.3% identified as female, 40.1% as male, and 1.6% preferred not to say. Campus-based sessions accounted for 71.2% of responses, and online or network testing links accounted for 28.8%. Campus and online sessions followed identical procedures and presented the same chat prototype and questionnaires; supplementary analyses found no main effect of testing channel and no Memory Type × Channel interaction on any focal outcome (all *p*≥0.18; Supplementary note S13). Session identifiers were not retained in the de-identified files, so intraclass correlations within recruitment sessions cannot be computed. Because the experiment program randomized conditions at the individual level within each session rather than assigning conditions by session, session-level clustering cannot confound the condition comparisons, although it could in principle affect standard errors. Supplementary Table S2 reports the composition and demographics of each study. We retain de-identified analysis files, the stimulus protocol, the data dictionary, analysis scripts, and exact statistics with the project materials. Raw screening logs are stored separately to protect participant privacy.

Sample sizes were determined by the number of eligible participants available within each study's scheduled recruitment window rather than by prospective power analysis. The experiment program assigned conditions in balanced blocks, and responses excluded by the screening rules were replaced by continued recruitment within the same window to keep the design cells balanced. This procedure explains why the retained cells are near-equal in Studies 1, 2, 5, and 6 and equal across the second factor in the factorial designs of Studies 3 and 4. Recruitment was never conditioned on outcome data: no interim analyses were conducted, and no study was stopped or extended based on observed effects. Achieved-sample power bounds for the original experimental test family are reported in the Supplementary Table S10.

**Figure 1:**
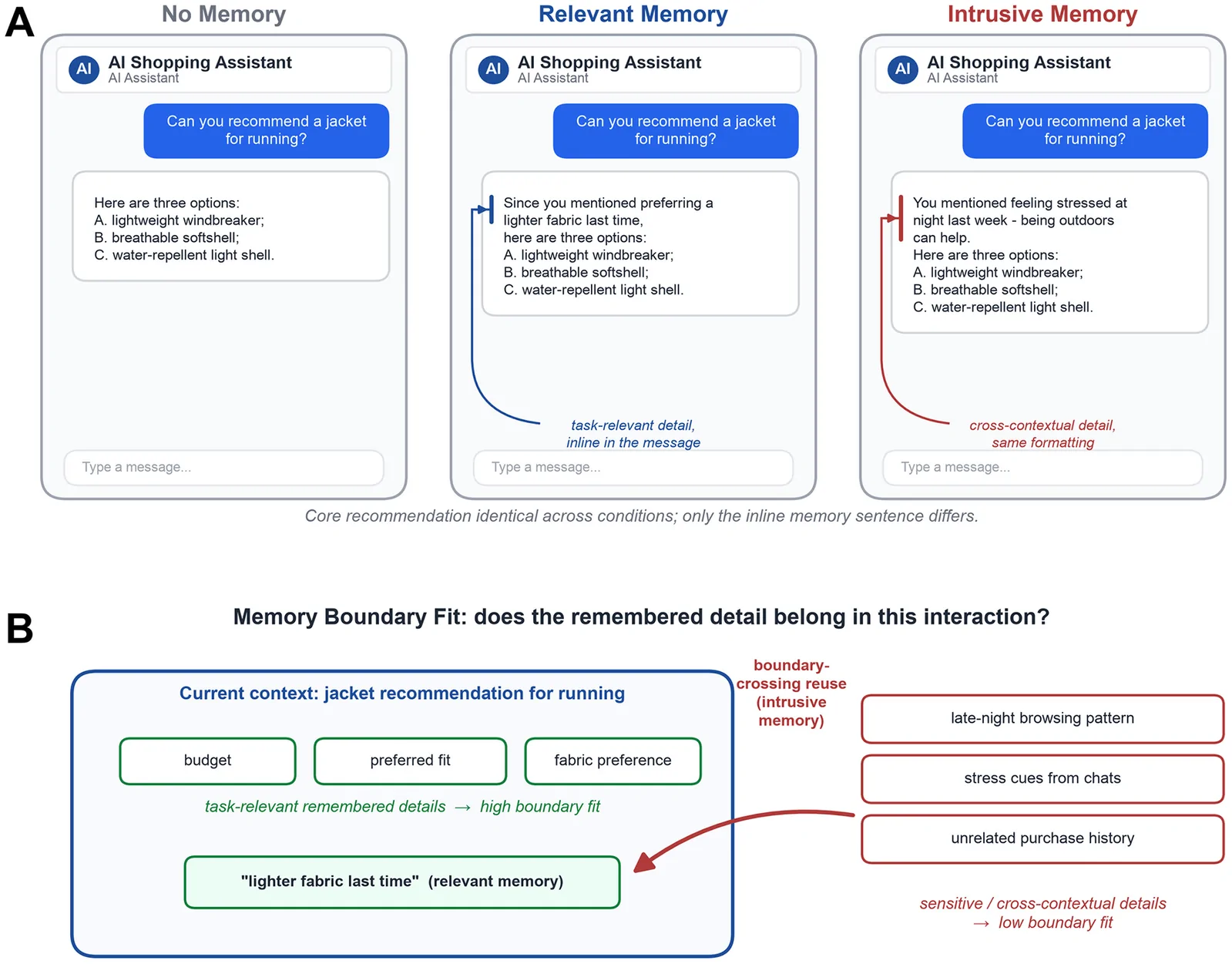
Schematic of the memory manipulation and boundary-fit logic. **(A)** Example replies in the no-memory, relevant-memory, and intrusive-memory conditions for the same request. The remembered detail appears within the assistant's message, while the core recommendation remains identical across conditions. **(B)** The contextual-integrity boundary separating task-relevant memories from sensitive, cross-contextual content. Within each scenario and dashboard level, the memory-type conditions used identical message formatting and visual styling, while memory-related wording differed.

In each study, participants chatted in real time with a mobile prototype powered by Google's gemini-3-flash-preview large language model (Figure 1). The prototype was accessed via the official Google API in Google AI Studio during the March–June 2026 collection window. Because this was a preview build that the provider could update without a public version change, we cannot guarantee exact regeneration of the stimulus text. The logged replies and the compliance checks described below therefore document what participants actually received. The chat delivered the experimental stimulus; outcome measures were standard self-reports completed immediately afterward. Within each scenario, and in Study 4 within each dashboard level, the assigned memory cue was inserted programmatically into an otherwise fixed reply template via a conversation-prefix variable. Thus, the memory-type conditions differed only in the memory sentence, while the core recommendation remained unchanged within that scenario; the scenario factor in Study 3 and the dashboard factor in Study 4 introduced the additional differences described below. Decoding used a low temperature to limit generative variance (temperature = 0.1, top-p = 0.8, maximum 600 tokens). Fixed prompts and condition-specific few-shot constraints enforced this structure. Each session comprised the participant's opening free-text request, the assistant's focal reply carrying the manipulation, and up to one free-text follow-up question. The system instruction required follow-ups to be answered from the fixed option set, with no new memory information introduced. The conversation ended after the follow-up, and participants proceeded directly to the questionnaire without the option to continue chatting. Within each scenario and dashboard level, message formatting and visual styling were identical across memory-type conditions, and all replies were logged. An automated check verified that the fixed recommendation was appropriate for the assigned scenario, memory condition, and permitted reply length for every retained session. A second check confirmed that no memory reference appeared in the no-memory condition; every retained session passed both checks. Measures followed a fixed order in every study: the MBF items (where measured) were administered immediately after the chat and before the trust items; trust and purchase intention preceded the broader platform judgments, and downstream engagement and intention measures came last. In every indirect-association model, the intermediate appraisal was therefore measured before the outcomes but within the same session. The Supplementary materials contain the full system prompt, few-shot constraints, decoding settings, and example exchanges for each condition.

### 2.2 Design of the six studies

The no-memory condition presented a generic recommendation without reference to prior interaction. The relevant-memory condition referenced information that clearly fit the current task, such as budget, intended use, or comfort preference. The intrusive-memory condition referenced sensitive or cross-contextual information, such as late-night browsing patterns, stress cues, or unrelated prior behavior. We refer to the three complete assistant messages as cue packages; the no-memory package is the same message with no memory reference. These packages differed jointly in topical relevance, information sensitivity, potential perceived usefulness, information source, and implied surveillance. Two of these attributes differ from the others. Topical relevance and information source are constitutive of what we intended to manipulate: on the contextual account, a remembered detail fits an exchange partly because it bears on the current task and partly because of how the assistant came to know it. Sensitivity, potential usefulness, and implied surveillance, by contrast, are attributes we did not intend to vary but could not hold constant while producing a recognizably intrusive cue. Neither group can be separated from the others in these data. The experiments therefore estimate responses to the cue packages as presented rather than isolating contextual fit, or any other single cue attribute, as the causal feature. In contextual-integrity terms (Nissenbaum, 2010), the actors and exchange setting were held constant across memory-type conditions within each scenario and dashboard level; the manipulated memory cue varied the information attributes (task-relevant vs. sensitive or cross-contextual content) and the implied transmission principle (reuse of information within its disclosure context vs. carryover from another context). Study 3 separately changed the complete scenario, and Study 4 separately varied the presence of one specific set of controls for inspecting, editing, or deleting stored memories (Table 1).

**Table 1:** Overview of study designs.

Study	Objective	Design	Scenario domain	Main outcomes	Theoretical role
1	Test memory type effects	No memory/Relevant/Intrusive	Apparel (jacket)	Trust, purchase intention, continued use	Tests the basic benefit-risk pattern
2	Examine indirect association	Memory type with measured MBF appraisal	Apparel (jacket)	MBF, trust	Estimates a same-session indirect association
3	Compare complete scenarios (exploratory)	Memory type × scenario	Apparel vs. sleep health	Trust	Does not isolate information sensitivity
4	Evaluate a specific dashboard	Memory type × inspect-edit-delete dashboard	Apparel (jacket)	Trust, MBF	Tests only the dashboard implementation presented
5	Compare engagement types	Memory type	Apparel (jacket)	Transactional, data-sharing, relational engagement	Compares MBF associations across engagement outcomes
6	Test downstream intentions	Memory type with serial indirect-association model	Apparel (jacket)	Privacy trust, loyalty intention, opt-in intention	Estimates same-session indirect associations with downstream intentions

Studies 1, 2, and 4–6 used an apparel scenario. Participants requested a jacket recommendation, and the assistant returned a fixed set of three options. Study 3 crossed memory type with scenario: apparel vs. sleep health. The sleep-health scenario used a parallel recommendation structure and recommended a sleep-monitoring ring, a smart white-noise machine, and melatonin drops. We selected the domains a priori because we expected them to differ in information sensitivity, consistent with evidence that health-related contexts are highly sensitive (Longoni et al., 2019). However, the scenarios also differed in product, recommendation content, cue wording, and other attributes, so Study 3 is presented as an exploratory comparison of two complete scenarios rather than as an isolated manipulation of sensitivity.

Studies 1 and 2 examined the basic memory-cue pattern, and Study 2 also examined same-session indirect associations involving MBF. Study 3 then compared the pattern across two complete scenarios, and Study 4 tested the specific inspect-edit-delete dashboard. Studies 5 and 6 extended the analysis to additional engagement outcomes, including privacy trust, loyalty intentions, and opt-in intentions.

### 2.3 Measures

In the six experiments, each focal construct was computed as the mean of three seven-point items. The outcome measures were adapted from established scales in electronic-commerce trust, personalization, and information-privacy research (McKnight et al., 2002; Komiak and Benbasat, 2006; Pavlou, 2003; Culnan and Armstrong, 1999; Malhotra et al., 2004), whereas the three MBF items were newly written for this research, based on the contextual-integrity account. Trust was measured in Studies 1–4; Study 1 also measured purchase intention and continued use. Study 5 measured transactional, data-sharing, and relational engagement. Study 6 measured privacy trust, loyalty intention, opt-in intention, and platform trust. MBF was measured in Studies 1, 2, and 4–6. Items were translated into Simplified Chinese and checked by translation and back-translation (Brislin, 1970). Supplementary note S14 documents the administration format, response anchors, scoring rules, presentation order, the source or theoretical basis for each measure, the administered wording with verified back-translation, and item-level psychometric evidence where retained data permit. MBF comprised three seven-point items assessing whether the remembered information was relevant, appropriate, and acceptable within the current interaction. The experimental analysis files retain MBF as a composite score. A supplemental validation sample (*N* = 234) completed the three MBF items alongside the nine-item IUIPC privacy-concern measure and three-item measures of relational warmth, surveillance concern, and trust. In this sample, MBF showed acceptable internal consistency (α = 0.76). Because a three-indicator one-factor model is just-identified, its standardized loadings follow in closed form from the inter-item correlations (0.62, 0.88, 0.66), computed from that common-factor solution rather than from a principal-component solution; composite reliability was CR = 0.77 and average variance extracted was AVE = 0.53. A five-factor CFA fit the data well ($\chi_{\left(179\right)}^{2}=197.85$, CFI = 0.990, RMSEA = 0.021, SRMR = 0.045) and reproduced the same loadings (0.58, 0.91, 0.66; AVE = 0.53, CR = 0.77). Discriminant-validity checks separated MBF from adjacent constructs, but by narrow margins: the maximum HTMT was 0.815, below the 0.85 criterion but close to it, and $\sqrt{\text{AVE}}=0.73$ exceeded the largest absolute composite correlation, |*r*| = 0.649 (with trust). The loadings are uneven, and both margins are modest, so these values support MBF only as an acceptable initial operationalization rather than as a strongly established scale. Trust reliability ranged from α = 0.86 to 0.88 across Studies 1–4. Reliability was acceptable for purchase intention (α = 0.86), continued use (α = 0.85), and transactional engagement (α = 0.86). Data-sharing and relational engagement each had α = 0.87. Privacy trust (α = 0.87), loyalty intention (α = 0.83), opt-in intention (α = 0.86), and platform trust (α = 0.85) showed similar reliability.

MBF differs conceptually from usefulness, general privacy concern, and control. Usefulness concerns whether remembered information supports the current task. Privacy concern captures broader worries about information collection and misuse, whereas control concerns the ability to govern stored information. The core recommendation remained constant across memory-type conditions within each scenario, while the presence and content of the memory cue varied. Study 2 measured relational warmth and surveillance concern, and the supplemental validation sample included IUIPC privacy concern, warmth, surveillance concern, and trust. In each sample, MBF explained approximately 20 percentage points of additional variance in trust beyond the adjacent appraisals measured in that sample (Supplementary note S15). Because MBF and trust were self-reported in the same session, this increment reflects a concurrent association and is inflated to an unknown degree by common-method variance. The studies did not directly measure perceived usefulness, creepiness, intrusiveness, information relevance, or perceived norm violation. Therefore, distinctions between MBF and these unmeasured appraisals remain conceptual rather than empirically established; Supplementary note S16 outlines each distinction and the study needed to test it.

### 2.4 Analytic strategy

We formulated all six hypotheses before data collection, but we did not preregister the studies. We therefore describe their designated tests, summarized in Table 2, as confirmatory in intent; this label concerns the timing of the expectations, not the strength of causal interpretation. Study 3 is an exception in interpretation: although motivated by an *a priori* sensitivity expectation, its bundled scenario manipulation does not isolate sensitivity, so we treat its interaction only as an exploratory comparison of the complete apparel and sleep-health scenarios. Auxiliary outcomes, construct correlations not designated for H5, competitive intermediate-appraisal comparisons, additional indirect-association models, and auxiliary contrasts are treated as exploratory. One-way and factorial ANOVAs tested experimental effects on the outcome measures, and we reported partial eta-squared ($\eta_{p}^{2}$) throughout. Values near 0.01, 0.06, and 0.14 were interpreted as small, medium, and large, respectively. We followed significant omnibus effects with Tukey HSD comparisons. Assumption checks for every experimental test in the cross-study multiplicity family (Levene's test for variance homogeneity; residual skewness, kurtosis, and Shapiro–Wilk tests) are reported in Supplementary note S13. Homogeneity held in every case except Study 3, whose interaction and expectation-aligned contrast were therefore re-estimated with heteroscedasticity-robust (HC3) standard errors and remained significant. Separately from the heterogeneous hypothesis-specific tests summarized in Table 2, a Holm–Bonferroni adjustment was applied to the original one-experimental-test-per-study family listed in Supplementary note S13. Every experimental effect in that family that was significant without adjustment remained significant after adjustment (largest adjusted *p* = 0.017), and the Study 4 interaction remained non-significant. This is a robustness check for the six-study experimental family, not a multiplicity correction for every coefficient or indirect association reported. Pairwise comparisons within each ANOVA were Tukey-adjusted. We report condition means with standard deviations and provide Tukey-adjusted pairwise differences and 95% confidence intervals in Supplementary Table S8. Ordinary least squares regression was used to estimate regression-based indirect-association models, conventionally termed mediation and serial-mediation models. We evaluated indirect associations using 95% percentile bootstrap confidence intervals from 5,000 resamples; intervals excluding zero were treated as statistically reliable. Because memory type is categorical, the primary models used two dummy variables, with no memory as the reference category, yielding separate relative indirect associations for relevant and intrusive memory (Hayes and Preacher, 2014). Ordinal-coded models (1 = intrusive, 2 = no memory, 3 = relevant) are reported only as supplementary summaries (Supplementary Tables S7, S9), because the no-memory condition presents no remembered content to evaluate and we do not interpret its MBF score as an intermediate level of fit. When an ordinal code is used, we report the dummy-coded and relevant-vs.-intrusive re-estimations alongside it. For the same reason, we additionally estimated the Study 2 and Study 6 models on the relevant-vs.-intrusive subsample alone, in which every participant rated an actual memory cue, and the intermediate appraisal therefore has an unambiguous referent in each cell (Supplementary Table S9b). Randomization supports the causal interpretation of condition assignment but does not establish the mediator-to-outcome paths or the ordering among same-session self-reports. For the original expectations in H3 and H4, we also report the difference-in-differences contrast (the intrusive-memory penalty across the two scenarios or dashboard levels) alongside the omnibus interaction (Supplementary note S13). Achieved-sample power analyses report the minimum detectable effect with 80% power at α = 0.05 for each experimental test in the six-study family (Supplementary Table S10). For non-significant experimental results, we report Bayes factors comparing the null and alternative models under two conventional default priors, because the strength of evidence for a null is prior-dependent and the two defaults diverge sharply in these designs. The first is the BIC approximation BF_01_ = exp[(BIC_*alt*_−BIC_*null*_)/2] (Wagenmakers, 2007), computed from the fitted OLS models of the corresponding ANOVA (null model excluding, alternative model including the focal term). This approximation corresponds to a unit-information prior, that is, a *g*-prior with *g* = *n*; because that prior is very diffuse, it imposes a large penalty on the alternative and is markedly biased toward the null at these sample sizes. The second is the Jeffreys–Zellner–Siow (JZS) mixture-of-*g*-priors Bayes factor computed from the same nested *F* test (Liang et al., 2008), which places a Cauchy prior on the standardized effect and belongs to the same family as the defaults implemented in common Bayesian ANOVA software (Rouder et al., 2012). Because that family is indexed by a prior scale, we verified that the conclusion does not depend on the scale chosen (Supplementary Table S12). We report the BIC value first for continuity with the wider literature, but we base our interpretation on the more conservative JZS value, under which none of the present nulls receives more than anecdotal-to-moderate support (Supplementary Table S12). We therefore treat these results as consistent with no effect of the size each design could detect (Supplementary Table S10), not as positive evidence that the effect is absent. Analyses were conducted in Python 3 using pandas, SciPy, and statsmodels. Analysis scripts are available from the corresponding author upon reasonable request.

**Table 2:** Summary of focal results across the six studies.

Study	*N*	Focal effect	Test statistic	Outcome
1	264	Memory type → trust	*F*_(2, 261)_ = 5.96, *p* = 0.003, $\eta_{p}^{2}=0.04$	H1 partially supported: relevant-memory benefit; purchase and intrusive-memory penalty not significant
2	289	MBF indirect-association model (dummy-coded)	Relative indirect associations = 0.33, 95% CI (0.18, 0.51) (relevant) and −0.42, 95% CI (−0.60, −0.27) (intrusive)	Consistent with H2 as an association; no causal mediation claim
3	410	Memory × complete scenario comparison	*F*_(2, 404)_ = 4.82, *p* = 0.009, $\eta_{p}^{2}=0.02$; penalty contrast Δ = 0.54, *p* = 0.031	H3 not directly tested; exploratory scenario comparison
4	516	Memory × specific dashboard	*F*_(2, 510)_ = 0.54, *p* = 0.584, $\eta_{p}^{2}=0.002$; penalty contrast Δ = 0.16, *p* = 0.407	H4 not supported for this dashboard; no general control claim
5	318	MBF association: data-sharing vs. transactional engagement	Difference in dependent *r* = 0.35, 95% CI (0.20, 0.49); difference-score *F*_(2, 315)_ = 5.78, *p* = 0.003	H5 supported for data-sharing vs. transactional, but not vs. relational engagement
6	343	Serial indirect associations with loyalty and opt-in intentions (dummy-coded)	Relative serial indirect associations = 0.14, 95% CI (0.08, 0.21) (relevant) and −0.15, 95% CI (−0.23, −0.09) (intrusive), both outcomes	Consistent with H6 for same-session intention outcomes

Indirect associations are reported with 95% percentile bootstrap confidence intervals (5,000 resamples). Relative indirect associations use no memory as the reference category.

### 2.5 Manipulation checks

Supplementary Table S3 reports the manipulation checks. Across all studies measuring MBF, scores were highest for relevant memory and lowest for intrusive memory, with the no-memory condition falling between them (all *p* < 0.001). This ordering indicates that the complete cue packages shifted the measured contextual-appropriateness appraisal in the intended direction. Because no remembered content is present in the no-memory condition, we interpret its MBF score as a neutral reference point rather than as an intermediate level of fit. Because MBF served as both the manipulation check and the intermediate variable in the indirect-association models, these checks are not independent evidence of a psychological mechanism. We therefore give primary weight to the randomized relevant-vs.-intrusive cue-package contrast.

## 3 Results

### 3.1 Study 1: memory type and trust

**Figure 2:**
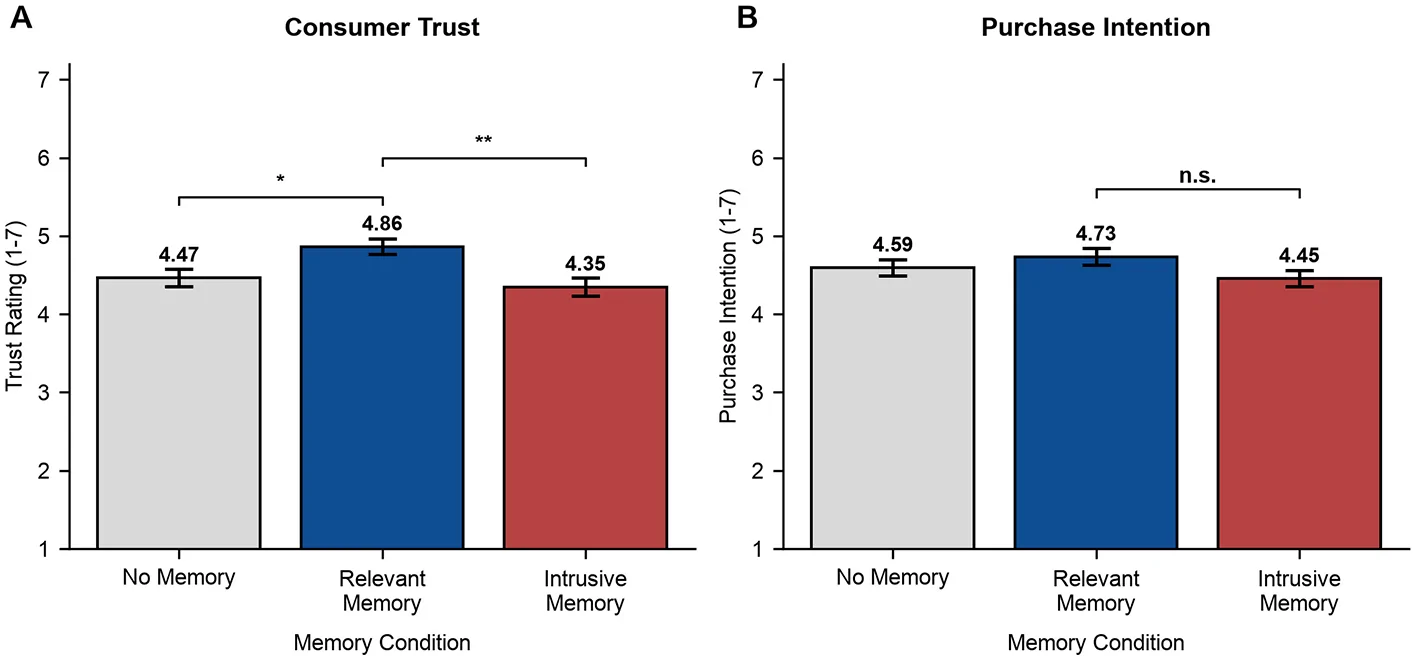
Study 1. **(A)** Trust and **(B)** purchase intention by memory type. Relevant memory increased trust relative to both other conditions; intrusive and no memory did not differ significantly. The omnibus effect on purchase intention was not significant, so the pairwise comparison shown in **(B)** is descriptive only. Brackets mark Tukey HSD pairwise comparisons (*p < 0.05, **p < 0.01; ns, not significant). Error bars show ±1 standard error of the mean.

A one-way ANOVA showed that memory type affected trust, *F*_(2, 261)_ = 5.96, *p* = 0.003, $\eta_{p}^{2}=0.04$. Mean trust was 4.86 under relevant memory (SD = 0.93), 4.47 under no memory (SD = 1.06), and 4.35 under intrusive memory (SD = 1.11; Figure 2). Tukey HSD comparisons showed that relevant memory exceeded no memory [diff = 0.40, 95% CI (0.03, 0.76), *p* = 0.028] and intrusive memory [diff = 0.51, 95% CI (0.14, 0.89), *p* = 0.003]. Intrusive and no memory did not differ [diff = 0.12, 95% CI (−0.25, 0.48), *p* = 0.736]. The results for continued use were similar, *F*_(2, 261)_ = 3.98, *p* = 0.020, $\eta_{p}^{2}=0.03$. Relevant memory (*M* = 4.82, SD = 0.99) exceeded no memory (*M* = 4.46, SD = 0.98; *p* = 0.040) and intrusive memory (*M* = 4.45, SD = 0.96; *p* = 0.040), while the latter two conditions did not differ (*p* = 0.999). Purchase intention had the same ordering, but the omnibus effect was not significant, *F*_(2, 261)_ = 1.76, *p* = 0.175, $\eta_{p}^{2}=0.01$. Bayes factors were sensitive to the prior: the BIC approximation favored the no-effect model (BF_01_ = 45.2), but the JZS default did not distinguish the two models (BF_01_ = 1.22). The observed $\eta_{p}^{2}=0.01$ also falls below the smallest effect this study could detect with 80% power ($\eta_{p}^{2}=0.036$; Supplementary Table S10), so the result is uninformative rather than evidence of absence (*M*_*relevant*_ = 4.73, SD = 0.98; *M*_*no*_ = 4.59, SD = 1.00; *M*_*intrusive*_ = 4.45, SD = 0.97). H1 was therefore partially supported. Relevant memory increased trust and continued use, but purchase intention and the intrusive-to-no-memory contrast were not significant.

### 3.2 Study 2: memory type, MBF, and trust

Study 2 replicated the omnibus effect of memory type on trust, *F*_(2, 286)_ = 19.86, *p* < 0.001, $\eta_{p}^{2}=0.12$, although the pairwise results differed from Study 1. Trust was lower under intrusive memory (*M* = 3.59, SD = 1.03) than under no memory [*M* = 4.17, SD = 1.00; diff = 0.59, 95% CI (0.24, 0.93), *p* < 0.001]. Trust was also lower under intrusive than relevant memory (*M* = 4.49, *SD* = 1.00; diff = 0.90, *p* < 0.001). The advantage of relevant over no memory was not significant (diff = 0.31, *p* = 0.076). Both studies produced the ordering relevant > no memory > intrusive. The reliable contrast reflected the benefit in Study 1 and the penalty in Study 2.

We next estimated a dummy-coded indirect-association model involving MBF, with no memory as the reference category (Hayes and Preacher, 2014). Relevant memory increased MBF (*a*_*rel*_ = 0.71, *p* < 0.001) and intrusive memory decreased it (*a*_*int*_ = −0.91, *p* < 0.001); MBF was associated with trust after adjustment for condition (*b* = 0.47, *p* < 0.001). The relative indirect association through MBF was 0.33 [95% bootstrap CI (0.18, 0.51)] for relevant vs. no memory and −0.42 [95% CI (−0.60, −0.27)] for intrusive vs. no memory; the corresponding relative direct associations were not significant (*p* = 0.910 and *p* = 0.220). A supplementary ordinal-coded summary model produced the same pattern [indirect association = 0.38, 95% CI (0.28, 0.49); direct association *p* = 0.327; Supplementary Table S9]. Because the no-memory condition presents no remembered content to evaluate, we also estimated the model on the relevant-vs.-intrusive subsample alone (*n* = 193), in which every participant judged an actual memory cue. Relevant memory raised MBF relative to intrusive memory (*a* = 1.61, SE = 0.15, *p* < 0.001), MBF was associated with trust (*b* = 0.44, SE = 0.06, *p* < 0.001), and the indirect association was 0.71 [95% bootstrap CI (0.49, 0.96)]; the direct association was not significant (*c*′ = 0.19, *p* = 0.254; Supplementary Table S9b). These results were consistent with H2 as a same-session statistical association. They do not establish that MBF causally transmitted the effect of condition to trust.

The auxiliary measures allowed us to compare MBF with relational warmth and surveillance concern. Relevant memory produced the highest warmth (*M* = 4.97) and lowest surveillance concern (*M* = 2.52); intrusive memory produced the reverse pattern (*M*_*warmth*_ = 3.62; *M*_*surveillance*_ = 4.34), *F*_(2, 286)_ = 52.87 and 95.17, respectively, both *p* < 0.001. MBF correlated positively with warmth (*r* = 0.42) and trust (*r* = 0.57), and negatively with surveillance concern (*r* = −0.55; Supplementary Table S5). In the supplemental validation sample, a five-factor CFA separated MBF from IUIPC, warmth, surveillance concern, and trust (CFI = 0.990, RMSEA = 0.021, SRMR = 0.045). In a competitive indirect-association model, MBF retained a specific indirect association (0.38, 95% CI [0.27, 0.50]), whereas the intervals for warmth and surveillance concern included zero; because a difference in significance is not a significant difference, we also bootstrapped the contrasts directly, and MBF's specific indirect association exceeded that of warmth (difference = 0.39, 95% CI [0.24, 0.55]) and of surveillance concern [difference = 0.40, 95% CI (0.23, 0.58)], which did not differ from each other (Supplementary Table S7). The same pattern holds when the competitive model is re-estimated with dummy codes or on the relevant-vs.-intrusive subsample (Supplementary Table S7). This comparison is associational and does not establish MBF as the causal mechanism.

### 3.3 Study 3: exploratory apparel–sleep-health scenario comparison

**Figure 3:**
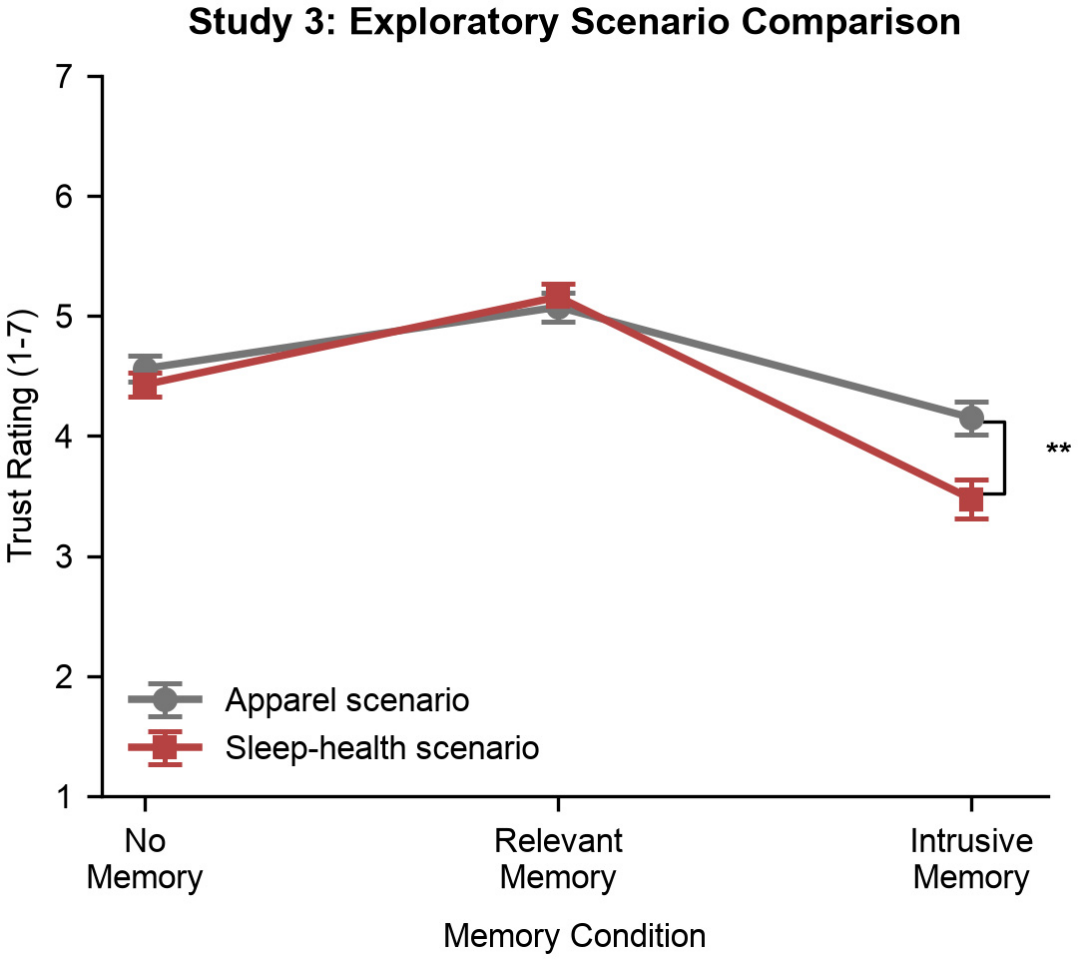
Study 3 exploratory scenario comparison. The complete sleep-health scenario produced a larger intrusive-memory trust penalty than the apparel scenario. Product context, recommendation content, cue wording, and other attributes changed together, so the interaction does not isolate information sensitivity. ^**^p < 0.01. Error bars show +/− 1 standard error of the mean.

Study 3 compared whether memory-type effects differed between the complete apparel and sleep-health scenarios. The Memory Type × Scenario interaction was significant, *F*_(2, 404)_ = 4.82, *p* = 0.009, $\eta_{p}^{2}=0.02$. Under intrusive memory, trust was lower in the sleep-health scenario (*M* = 3.48, SD = 1.34) than in the apparel scenario (*M* = 4.15, SD = 1.13). Trust under no memory and relevant memory changed little across scenarios (Figure 3). Relative to no memory, the intrusive-memory penalty was 0.41 in the apparel scenario [95% CI (−0.0004, 0.8202), *p* = 0.0503] and 0.95 in the sleep-health scenario [95% CI (0.53, 1.38), *p* < 0.001]. The difference-in-differences contrast between the two complete scenarios was significant [Δ = 0.54, SE = 0.25, *t*_(404)_ = 2.17, *p* = 0.031, 95% CI (0.05, 1.04)]. Because Levene's test indicated unequal cell variances, the interaction and contrast were re-estimated with heteroscedasticity-robust (HC3) standard errors and remained significant (robust interaction *F* = 4.02, *p* = 0.018; robust contrast *z* = 2.06, *p* = 0.039; Supplementary note S13). We treat this as an exploratory scenario comparison, not as a direct test of H3. Product context, information sensitivity, recommendation content, cue wording, potential usefulness, and other scenario characteristics changed together. The result therefore establishes only that the complete sleep-health package carried a larger intrusive-memory penalty than the complete apparel package; it does not identify information sensitivity as the responsible attribute. A valid test of H3 would hold cue content, source, plausibility, and recommendation structure as closely matched as possible while varying sensitivity alone.

### 3.4 Study 4: the specific editable dashboard

**Figure 4:**
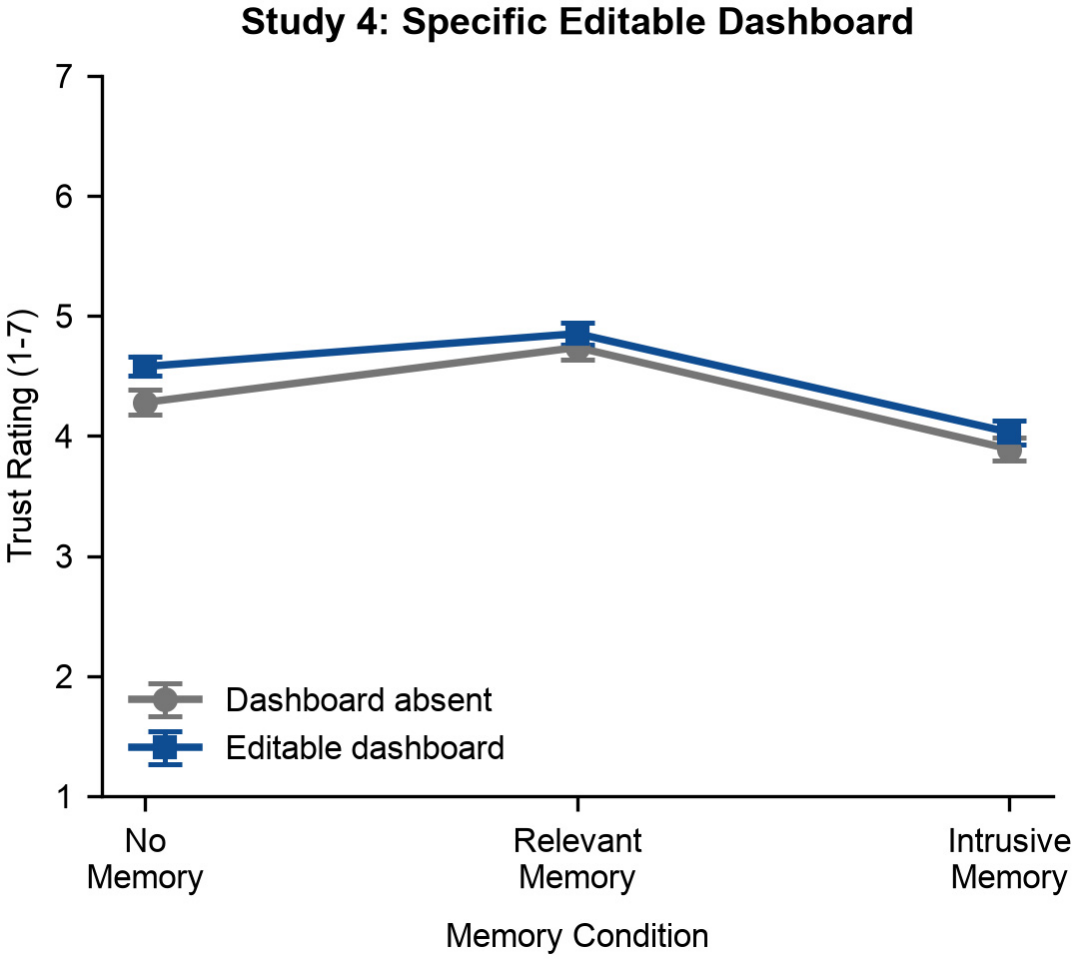
Study 4. Trust by memory type and availability of the specific inspect-edit-delete dashboard tested. The dashboard increased average trust (*p* = 0.019), but the Memory Type × Dashboard interaction was not significant. Thus, this dashboard did not selectively reduce the intrusive-memory penalty; the result does not establish that user control is generally ineffective. Error bars show ±1 standard error of the mean.

Study 4 evaluated whether the inspect-edit-delete dashboard tested here reduced the intrusive-memory penalty. A 3 × 2 ANOVA found a main effect of memory type on trust, *F*_(2, 510)_ = 36.91, *p* < 0.001, $\eta_{p}^{2}=0.13$. All three Tukey comparisons were significant, including the intrusive-to-no-memory penalty [diff = 0.47, 95% CI (0.24, 0.70), *p* < 0.001]. The dashboard also had a main effect on trust, *F*_(1, 510)_ = 5.54, *p* = 0.019, $\eta_{p}^{2}=0.011$. Mean trust was 4.49 when the dashboard was present (SD = 0.91) and 4.30 when it was absent (SD = 1.00). The Memory Type × Dashboard interaction was not significant, *F*_(2, 510)_ = 0.54, *p* = 0.584, $\eta_{p}^{2}=0.002$. The difference-in-differences contrast comparing the intrusive-memory penalty with and without this dashboard was likewise not significant [Δ = 0.16, SE = 0.19, *t*_(510)_ = 0.83, *p* = 0.407, 95% CI (−0.22, 0.54)]. A BIC-approximation Bayes factor favored the additive model over the interaction model (BF_01_ = 299.5), but this figure should not be read as decisive: under the JZS default the same comparison yields only BF_01_ = 2.26, and the observed $\eta_{p}^{2}=0.002$ is far below the smallest interaction this design could detect ($\eta_{p}^{2}=0.018$; Supplementary Table S10). The intrusive-memory means were 4.03 with the dashboard and 3.89 without it (Figure 4). The interaction for MBF was also null (*F*_(2, 510)_ = 1.74, *p* = 0.177; BF_01_ = 89.4 by the BIC approximation but 1.23 under the JZS default). Mean MBF was 4.14 with the dashboard and 4.30 without it, *F*_(1, 510)_ = 3.77, *p* = 0.053. Here the two priors disagree in direction: the BIC approximation favors no dashboard main effect on MBF (BF_01_ = 3.45), whereas the JZS default slightly favors an effect (BF_01_ = 0.65, i.e., BF_10_ = 1.54). We therefore treat this comparison as undetermined. We note that the descriptive direction is opposite to the H4 prediction: MBF was nominally lower, not higher, when the dashboard was present. We do not interpret this difference, which is not statistically reliable, but it gives no support to the expectation that visible controls raise the perceived fit of a specific remembered detail. This dashboard raised trust on average but did not selectively reduce the intrusive-memory penalty or produce the MBF improvement specified in H4. H4 was therefore not supported for the implementation tested. Because we did not include a perceived-control manipulation check, this result is limited to the dashboard implementation, presentation, and timing tested here; it leaves the general form of H4 untested and does not show that user control is generally ineffective.

### 3.5 Study 5: engagement type

Memory type affected data-sharing engagement, *F*_(2, 315)_ = 24.03, *p* < 0.001, $\eta_{p}^{2}=0.13$, and relational engagement, *F*_(2, 315)_ = 14.80, *p* < 0.001, $\eta_{p}^{2}=0.09$. It did not affect transactional engagement, *F*_(2, 315)_ = 1.95, *p* = 0.144, $\eta_{p}^{2}=0.01$. The BIC-approximation Bayes factor favored the no-effect model for transactional engagement (BF_01_ = 44.9), although the JZS default was equivocal (BF_01_ = 1.10), and all pairwise comparisons had *p*≥0.121. For data-sharing engagement, relevant memory exceeded no memory (*M* = 4.86, SD = 0.91, vs. *M*_*no*_ = 4.20, SD = 1.08; diff = 0.66, *p* < 0.001). Intrusive and no memory did not differ reliably (diff = 0.27, *p* = 0.118). Thus, the data-sharing result primarily reflected a relevant-memory benefit rather than a reliable intrusive-memory penalty. Relational engagement showed both a relevant-memory benefit (*p* = 0.010) and an intrusive-memory penalty (*p* = 0.032). H5 concerns the relative strength of MBF's associations with the two engagement types. The focal test therefore compared the two dependent correlations sharing MBF: the MBF association with data-sharing engagement (*r* = 0.30, *p* < 0.001) exceeded the association with transactional engagement (*r* = −0.05, *p* = 0.423), difference in *r* = 0.35, 95% CI [0.20, 0.49], where the confidence interval for the difference between the dependent correlations was obtained from a 5,000-resample percentile bootstrap (Supplementary Table S11). As a complementary experimental contrast, we regressed each participant's data-sharing vs. transactional difference score on memory type; this contrast was also significant, *F*_(2, 315)_ = 5.78, *p* = 0.003, $\eta_{p}^{2}=0.04$. MBF also correlated with relational engagement (*r* = 0.20, *p* < 0.001), and the data-sharing association did not reliably exceed the relational association [difference in *r* = 0.11, 95% CI (−0.03, 0.24), same bootstrap procedure]. These results supported H5 for the focal data-sharing vs. transactional contrast.

### 3.6 Study 6: privacy trust and downstream intentions

Memory type affected loyalty intention, *F*_(2, 340)_ = 9.77, *p* < 0.001, $\eta_{p}^{2}=0.05$, and opt-in intention, *F*_(2, 340)_ = 17.13, *p* < 0.001, $\eta_{p}^{2}=0.09$. For opt-in intention, both pairwise components were significant (relevant vs. no memory: diff = 0.31, *p* = 0.038; no memory vs. intrusive: diff = 0.43, *p* = 0.002). For loyalty intention, the relevant- vs.-intrusive difference was clear (diff = 0.56, *p* < 0.001), but neither comparison with no memory reached significance (benefit *p* = 0.055; penalty *p* = 0.085). In the primary dummy-coded serial indirect-association model (no memory as reference), relevant memory increased MBF (*a*_*rel*_ = 0.77, *p* < 0.001) and intrusive memory decreased it (*a*_*int*_ = −0.85, *p* < 0.001); MBF was associated with privacy trust (*d*_21_ = 0.42, *p* < 0.001), and privacy trust was associated with both loyalty intention (*b*_2_ = 0.43, *p* < 0.001) and opt-in intention (*b*_2_ = 0.44, *p* < 0.001). The relative serial indirect associations were 0.14 [95% CI (0.08, 0.21)] for relevant vs. no memory and −0.15 [95% CI (−0.23, −0.09)] for intrusive vs. no memory, for both outcomes (Supplementary Table S9). A supplementary ordinal-coded summary model gave equivalent estimates [0.15 for each outcome, 95% bootstrap CIs (0.10, 0.20)]. On the relevant-vs.-intrusive subsample (*n* = 227), the serial indirect association was 0.31 [95% CI (0.20, 0.44)] for loyalty intention, where the direct association was not significant (*c*′ = 0.07, *p* = 0.591), and 0.34 [95% CI (0.23, 0.47)] for opt-in intention, where a direct association remained (*c*′ = 0.39, *p* = 0.007; Supplementary Table S9b). The pattern was consistent with H6 for same-session intention outcomes. It does not establish the causal ordering of MBF, privacy trust, or the outcomes, nor does it establish longitudinal changes in realized loyalty behavior.

### 3.7 Synthesis across the six studies

The six studies build a cumulative rather than a repetitive case across three stages of evidence. Studies 1 and 2 established the memory-cue pattern, and Study 2 produced same-session indirect associations consistent with the proposed MBF account. Study 3 showed that the penalty differed across two complete scenario packages but did not identify sensitivity as the responsible attribute. Study 4 showed that the specific dashboard tested did not selectively attenuate the penalty. Studies 5 and 6 extended the analysis beyond immediate trust to data-sharing engagement and downstream intentions, with privacy trust included as an intermediate appraisal in the Study 6 same-session model.

The principal and most consistent finding across studies is the contrast between the complete relevant-memory and intrusive-memory cue packages. This contrast was reliable for trust in Studies 1–4, for data-sharing and relational engagement in Study 5, and for loyalty and opt-in intentions in Study 6; it was not reliable for Study 1 purchase intention or Study 5 transactional engagement, whose omnibus tests were also null (Table 3; Supplementary Table S8). Relevant memory was at least as favorable as no memory, which was at least as favorable as intrusive memory, for every outcome, but the reliable pairwise comparisons with no memory varied across studies. A relevant-memory benefit was reliable for Study 1 trust and continued use, both Study 3 scenarios, Study 4 trust, Study 5 data-sharing and relational engagement, and Study 6 opt-in intention. An intrusive-memory penalty was reliable for Study 2 trust, the Study 3 sleep-health scenario, Study 4 trust, Study 5 relational engagement, and Study 6 opt-in intention. Thus, both components were reliable simultaneously only for the Study 3 sleep-health scenario, Study 4 trust, Study 5 relational engagement, and Study 6 opt-in intention. The personalization benefit and the intrusion penalty should therefore not be regarded as uniformly established relative to no memory.

**Table 3:** Relevant-vs.-intrusive contrasts for the principal outcomes.

Study	Outcome	Relevant minus intrusive	Qualification
1	Trust; continued use	0.51, *p* = 0.003; 0.36, *p* = 0.040	Purchase intention was not reliable (0.28, *p* = 0.148)
2	Trust	0.90, *p* < 0.001	Relevant vs. no memory was not reliable (*p* = 0.076)
3	Trust: apparel; sleep health	0.92, *p* < 0.001; 1.68, *p* < 0.001	Complete scenarios differed on multiple attributes
4	Trust (pooled across dashboard)	0.83, *p* < 0.001	No selective attenuation by the tested dashboard
5	Data-sharing; relational engagement	0.94, *p* < 0.001; 0.74, *p* < 0.001	Transactional engagement was not reliable (0.28, *p* = 0.121)
6	Loyalty; opt-in intention	0.56, *p* < 0.001; 0.75, *p* < 0.001	Loyalty comparisons with no memory were not reliable

Positive differences indicate higher scores under the relevant-memory cue package. Pairwise *p* values are Tukey-adjusted.

Two qualifications limit what the principal contrast can carry. First, given the descriptive ordering relevant > no memory > intrusive, the relevant-vs.-intrusive comparison spans the two extremes and, by construction, is the largest of the three pairwise differences; it is correspondingly the best powered, so part of its greater consistency is a mechanical consequence of the ordering rather than independent evidence. Second, because it combines the two components, it is silent about their separate magnitudes: a reliable relevant-vs.-intrusive difference is compatible with a benefit alone, a penalty alone, or both. We report the decomposition above and in Supplementary Table S8 precisely so that this contrast is not read as establishing either component on its own. Table 2 reports each designated hypothesis test and outcome, and Table 4 describes the relations among constructs.

**Table 4:** Construct interpretation across studies.

Construct	Conceptual role	Distinction from adjacent constructs	Main studies
Memory boundary Fit	Fit between remembered content and the contextual boundary	Preliminary operationalization, narrower than general privacy concern and conceptually differentiated from task usefulness. In the competitive indirect-association model, only MBF retained a reliable indirect association when MBF, warmth, and surveillance concern were entered jointly, and direct bootstrap contrasts confirm that its specific indirect association exceeds each competitor's (Supplementary Table S7). The validation sample supports reliability and factorial separation from measured constructs (Supplementary Table S5). MBF explains Δ*R*^2^≈0.20 of additional variance in trust over the measured appraisals, though same-session self-report inflates this increment (Supplementary note S15). Empirical separation from usefulness, creepiness, intrusiveness, relevance, and norm violation remains untested (Supplementary note S16); no causal-mechanism claim is made	1, 2, 4–6; validation sample
Privacy trust	Confidence in the platform's information practices	Targets platform information handling rather than the fit of a single remembered item; included as an intermediate variable in the same-session serial indirect-association model	6
Surveillance concern	Concern that memory reflects monitoring	An affective monitoring appraisal rather than a contextual fit judgment; correlated with MBF (*r* = −0.55) but did not retain a reliable indirect association in the competitive model (Supplementary Table S7)	2
Memory control	Visible controls to inspect, edit, or delete memory	A specific interface affordance tested in Study 4, not evidence about user control generally	4
Data-sharing engagement	Willingness to authorize additional information use	A downstream behavioral intention, more sensitive to memory type than transactional engagement (Study 5 within-person contrast)	5
Loyalty intention	Intended continuation of the relationship with the interface	A same-session intention included downstream of privacy trust in an associational serial model	6

## 4 Discussion

Across the six studies, complete relevant-memory cue packages were generally evaluated more favorably than intrusive-memory cue packages, with no memory typically falling between them. This pattern is consistent with, but does not establish, a contextual account in which consumers evaluate remembered information in relation to the current interaction. General preferences for personalization, usefulness, less sensitive data use, or reduced surveillance may also contribute, as the packages varied in topical relevance, sensitivity, perceived usefulness, information source, and implied surveillance. The relevant-memory benefit and the intrusive-memory penalty relative to no memory varied across studies. The complete sleep-health scenario produced a larger intrusive-memory penalty than the apparel scenario, but the attribute responsible was not identified. Data-sharing engagement was more responsive than transactional engagement, primarily because relevant memory increased data-sharing engagement (Zhu et al., 2025). The specific dashboard tested increased average trust but did not selectively alter the effect of memory type. Throughout, the evidence concerns simulated claims of memory delivered in a single session rather than genuine retrieval of previously disclosed information.

### 4.1 Theoretical contributions

The findings make three related but preliminary theoretical contributions. First, they connect simulated memory-cue packages with consumer trust and engagement outcomes. This extends agent-memory research that has concentrated on usability and privacy expectations (Jones et al., 2025; Zhang et al., 2025; Mekioussa Malki, 2026). Second, MBF offers an interaction-specific framing of the personalization-privacy paradox (Awad and Krishnan, 2006; Xu et al., 2011; Norberg et al., 2007). The relative weight of personalization benefit and privacy risk may depend on whether consumers regard a particular use of memory as appropriate in the exchange. This interpretation accords with evidence that privacy judgments change with context and framing (Acquisti et al., 2015; Adjerid et al., 2019). It also responds to calls to study the paradox in specific contexts (Mao et al., 2026).

Third, MBF provides a preliminary measurable consumer appraisal informed by contextual integrity (Nissenbaum, 2010). It offers one candidate account of why cross-context information reuse may be associated with lower trust, even when the recommendation is useful. Across Study 2 and the validation sample, MBF explained roughly twenty percentage points of additional variance in trust beyond the adjacent appraisals measured alongside it. Because all of these measures were self-reported in the same session, part of that increment reflects common-method variance rather than construct-specific information. This supports a limited measurement claim, not a claim of identifying a psychological mechanism. The present evidence does not separate MBF from perceived usefulness, creepiness, intrusiveness, information relevance, or norm violation, none of which were measured. This boundary is especially important because the cue packages also differed in topical relevance, sensitivity, potential perceived usefulness, information source, and implied surveillance. The framework may also connect memory design with consumer autonomy over personal data (André et al., 2018) and connected objects that retain data during ongoing use (Hoffman and Novak, 2018).

### 4.2 Design implications

The findings suggest that memory may affect the user-facing relationship and therefore should not be considered solely a backend capability. They motivate testing whether systems benefit from distinguishing memories that serve the current task from sensitive information carried over from another context, and from informing users why a recommendation appeared. Conservative defaults in health, finance, and identity-related settings remain a design proposition that requires direct evaluation with genuine persistent memory.

Study 4 provides evidence only about the specific dashboard implementation tested. It increased average trust but did not improve MBF or selectively reduce the intrusive-memory penalty. An inspect-edit-delete dashboard may signal procedural control without altering the judgment of a specific memory cue, but other forms, strengths, or timing of control may produce different results. Moreover, without a perceived-control manipulation check, we cannot determine whether participants experienced the interface as providing meaningful control. The null interaction therefore should not be interpreted as showing that editable control is generally ineffective. At most, the findings motivate treating memory selection and user control as complementary design questions for future testing. Prior research similarly finds that control and trust-building measures can reduce but not eliminate negative reactions to data collection (Aguirre et al., 2015; Tucker, 2014).

### 4.3 Managerial and policy implications

Because the evidence derives from brief simulated interactions in a single national context and relies predominantly on self-reported intentions, the following implications are offered as tentative directions rather than prescriptions. For managers, the results caution against assuming that greater memory visibility will always improve personalization. The findings motivate testing recency, relevance, user expectations, and memory governance in real-world systems. Trust and data-sharing measures may reveal costs that conversion rates alone miss. The larger penalty in the complete sleep-health scenario suggests that uniform memory policies may perform differently across categories, but the present study cannot support category-specific prescriptions or attribute the difference to sensitivity. Replication with matched memory cues is required.

Data protection rules that focus on collection and consent may not capture every harmful use. Information can be lawfully collected and later reused in ways that violate situational expectations. Regulators and platform auditors could assess contextual reuse alongside collection practices. Relevant criteria include memory persistence, default retention, and cross-application transfer. These policy directions are normatively motivated extensions of the findings rather than empirically tested prescriptions; the studies did not examine legal compliance, consent behavior, or real-world data flows.

### 4.4 Construct boundaries

The supplemental validation sample distinguished MBF from general privacy concern, surveillance concern, relational warmth, and trust (Supplementary Table S5). In Study 2, MBF retained a reliable specific indirect association when warmth and surveillance concern were entered into the same model, and bootstrap contrasts placed it above each of them (Supplementary Table S7). These results concern measured covariance and do not identify causal ordering.

Two-sample tests also allow a direct incremental-validity test against the adjacent appraisals measured concurrently (Supplementary note S15). In Study 2, entering MBF after relational warmth and surveillance concern increased the variance explained by trust from *R*^2^ = 0.13 to *R*^2^ = 0.33 (Δ*R*^2^ = 0.199, *F*_(1, 285)_ = 84.30, *p* < 0.001). In the validation sample, entering MBF after IUIPC privacy concern, warmth, and surveillance concern increased *R*^2^ from 0.26 to 0.46 (Δ*R*^2^ = 0.200, *F*_(1, 229)_ = 84.33, *p* < 0.001). In Study 2, reverse-order checks showed that warmth and surveillance concern each added only Δ*R*^2^ < 0.001 after MBF (both *p* = 0.854); the archived validation analysis reports the corresponding reverse-order check only for surveillance concern (Δ*R*^2^ = 0.001, *p* = 0.465). The primary incremental results indicate that MBF contributed information beyond the measured appraisal set in each sample, while leaving its distinction from the unmeasured alternatives unresolved.

These findings support MBF only as a preliminary, context-specific operationalization of remembered-content evaluation. They do not establish empirical separation from perceived usefulness, creepiness, intrusiveness, information relevance, or perceived norm violation because the studies did not measure these alternatives directly. Supplementary note S16 presents a systematic conceptual comparison of MBF with each of these neighbors, specifying for each the judgment it targets, the point at which it should diverge from MBF, and the measurement that would adjudicate between them. A remembered detail may be useful yet inappropriate, or unobjectionable yet of little value. Related research has examined boundary crossing in personalized advertising (Boerman et al., 2021) and creepiness in data-driven personalization (Tene and Polonetsky, 2014; Petrova et al., 2026). Communication privacy management (Petronio, 2002, 2013), expectancy-violation theory (Burgoon, 1993), and research on perceived intrusiveness (Edwards et al., 2002) each offer a candidate account of the same reaction. Discriminating MBF from these accounts requires measuring them alongside MBF, which the present studies did not do. The present manuscript therefore does not claim that MBF is an established or distinctive psychological mechanism; that question requires new data with matched manipulations, independent checks, and separate measurement occasions.

### 4.5 Limitations and future research

The indirect-association models used measures collected within the same session, so the intermediate-to-outcome paths remain correlational and may reflect common-method variance, reverse ordering, consistency bias, or conceptual overlap, even though MBF was measured before the outcomes. MBF also served as both the manipulation check and the intermediate variable, so the indirect associations may partly reflect semantic correspondence between the cue packages and the MBF items. In the no-memory condition, participants evaluated MBF despite the absence of remembered content; the primary models therefore used dummy coding, and we do not interpret that condition's MBF score as an intermediate level of fit. The complete cue packages differed in topical relevance, information sensitivity, perceived usefulness, information source, and implied surveillance. Therefore, the design cannot isolate contextual fit, or any other single attribute, as the causal feature. In Study 3, product context, recommendation content, and cue wording changed together, preventing attribution of the interaction to information sensitivity. Construct validation was conducted in a supplemental sample rather than within each experimental sample. In that sample, the three-item scale met conventional reliability and discriminant-validity thresholds only by modest margins, with uneven item loadings. Its separation from trust is the weakest: after attenuation correction, the two constructs share roughly two-thirds of their reliable variance, so MBF is best treated as a distinguishable but closely related antecedent of trust rather than a clearly independent construct. The scale therefore requires refinement before it can carry heavier inferential weight. Future studies should use orthogonal matched manipulations, independent manipulation checks, separated measurement occasions, and behavioral permission decisions. The present studies measured willingness to share data and opt-in intentions rather than actual choices.

Generalization is limited by recruitment at and around a single Chinese institution. Because participation was anonymous, we also cannot rule out that some individuals took part in more than one study. Privacy boundaries may vary across cultural and regulatory settings. Across the original one-experimental-test-per-study family, $\eta_{p}^{2}$ ranged from 0.002 to 0.13; the reliable effects ranged from 0.02 to 0.13 and were small to moderate. A single exposure should not be interpreted as producing a large behavioral change.

The studies examined brief interactions with text-based assistants and simulated memory cues. Participants had no established relationship with the assistant, and the study did not assess suspicion or cover-story credibility; some participants may have viewed the memory references as fabricated or inaccurate. Conclusions therefore pertain to responses to simulated memory claims rather than to persistent memory of genuinely disclosed information. Research using genuinely retained information across repeated sessions is needed to examine memory accumulation, decay, and user-vs. system-initiated retention.

## 5 Conclusion

Across six controlled single-session chat experiments using simulated memory cues, the principal and most consistent finding was that complete relevant-memory cue packages produced more favorable trust-related outcomes than intrusive-memory cue packages. Comparisons with no memory varied across studies. The complete sleep-health scenario produced a larger intrusive-memory penalty than the apparel scenario, although multiple scenario characteristics changed together. Data-sharing engagement was more responsive than transactional engagement, primarily through a relevant-memory benefit. The specific editable dashboard tested raised average trust but did not selectively reduce the intrusive-memory penalty or improve MBF.

Together, the findings support contextual appropriateness as a candidate design consideration alongside retrieval accuracy, usefulness, and user control. The evidence remains limited by short interactions, simulated composite cues, and predominantly self-reported outcomes. Whether the same pattern holds for genuine persistent memory, repeated interactions, behavioral choices, or other forms of user control remains to be established.

## Data Availability

The datasets presented in this study can be found in online repositories. The names of the repository/repositories and accession number(s) can be found below: https://github.com/RunhuaHuang/aimemory.

## References

[B1] Acquisti A. Brandimarte L. Loewenstein G. (2015). Privacy and human behavior in the age of information. Science 347, 509–514. doi: 10.1126/science.aaa1465 25635091

[B2] Acquisti A. Taylor C. Wagman L. (2016). The economics of privacy. J. Econ. Lit. 54, 442–492. doi: 10.1257/jel.54.2.442

[B3] Adjerid I. Acquisti A. Loewenstein G. (2019). Choice architecture, framing, and cascaded privacy choices. Manage. Sci. 65, 2267–2290. doi: 10.1287/mnsc.2018.3028

[B4] Aguirre E. Mahr D. Grewal D. de Ruyter K. Wetzels M. (2015). Unraveling the personalization paradox: the effect of information collection and trust-building strategies on online advertisement effectiveness. J. Retail. 91, 34–49. doi: 10.1016/j.jretai.2014.09.005

[B5] André Q. Carmon Z. Wertenbroch K. Crum A. Frank D. Goldstein W. . (2018). Consumer choice and autonomy in the age of artificial intelligence and big data. Cust. Needs Solut. 5, 28–37. doi: 10.1007/s40547-017-0085-8

[B6] Awad N. F. Krishnan M. S. (2006). The personalization privacy paradox: an empirical evaluation of information transparency and the willingness to be profiled online for personalization. MIS Q. 30, 13–28. doi: 10.2307/25148715

[B7] Bano S. Sarfraz U. Salameh A. A. Jan A. (2022). COVID-19 paradox: the role of privacy concerns and ad intrusiveness on consumer's attitude toward app usage behavior. Front. Psychol. 13:836060. doi: 10.3389/fpsyg.2022.836060 35707643 PMC9191572

[B8] Blut M. Wang C. Wünderlich N. V. Brock C. (2021). Understanding anthropomorphism in service provision: a meta-analysis of physical robots, chatbots, and other ai. J. Acad. Market. Sci. 49, 632–658. doi: 10.1007/s11747-020-00762-y

[B9] Boerman S. C. Kruikemeier S. Bol N. (2021). When is personalized advertising crossing personal boundaries? how type of information, data sharing, and personalized pricing influence consumer perceptions of personalized advertising. Comput. Hum. Behav. Rep. 4:100144. doi: 10.1016/j.chbr.2021.100144

[B10] Brislin R. W. (1970). Back-translation for cross-cultural research. J. Cross Cult. Psychol. 1, 185–216. doi: 10.1177/135910457000100301

[B11] Burgoon J. K. (1993). Interpersonal expectations, expectancy violations, and emotional communication. J. Lang. Soc. Psychol. 12, 30–48. doi: 10.1177/0261927X93121003

[B12] Castelo N. Bos M. W. Lehmann D. R. (2019). Task-dependent algorithm aversion. J. Market. Res. 56, 809–825. doi: 10.1177/0022243719851788

[B13] Culnan M. J. Armstrong P. K. (1999). Information privacy concerns, procedural fairness, and impersonal trust: an empirical investigation. Organ. Sci. 10, 104–115. doi: 10.1287/orsc.10.1.104

[B14] Dinev T. Hart P. (2006). An extended privacy calculus model for e-commerce transactions. Inf. Syst. Res. 17, 61–80. doi: 10.1287/isre.1060.0080

[B15] Edwards S. M. Li H. Lee J.-H. (2002). Forced exposure and psychological reactance: antecedents and consequences of the perceived intrusiveness of pop-up ads. J. Advert. 31, 83–95. doi: 10.1080/00913367.2002.10673678

[B16] Gefen D. Karahanna E. Straub D. W. (2003). Trust and tam in online shopping: an integrated model. MIS Q. 27, 51–90. doi: 10.2307/30036519

[B17] Glikson E. Woolley A. W. (2020). Human trust in artificial intelligence: review of empirical research. Acad. Manage. Ann. 14, 627–660. doi: 10.5465/annals.2018.0057

[B18] Hayes A. F. Preacher K. J. (2014). Statistical mediation analysis with a multicategorical independent variable. Br. J. Math. Stat. Psychol. 67, 451–470. doi: 10.1111/bmsp.12028 24188158

[B19] Hildebrand C. Bergner A. (2021). Conversational robo advisors as surrogates of trust: onboarding experience, firm perception, and consumer financial decision making. J. Acad. Market. Sci. 49, 659–676. doi: 10.1007/s11747-020-00753-z

[B20] Hoffman D. L. Novak T. P. (2018). Consumer and object experience in the internet of things: an assemblage theory approach. J. Consum. Res. 44, 1178–1204. doi: 10.1093/jcr/ucx105

[B21] John L. K. Acquisti A. Loewenstein G. (2011). Strangers on a plane: context-dependent willingness to divulge sensitive information. J. Consum. Res. 37, 858–873. doi: 10.1086/656423

[B22] Jones B. Stemmler K. Su E. Kim Y.-H. Kuzminykh A. (2025). “Users' expectations and practices with agent memory,” in Proceedings of the extended abstracts of the CHI conference on human factors in computing systems (New York, NY: ACM), 1–8. doi: 10.1145/3706599.3720158

[B23] Komiak S. Y. X. Benbasat I. (2006). The effects of personalization and familiarity on trust and adoption of recommendation agents. MIS Q. 30, 941–960. doi: 10.2307/25148760

[B24] Komiak S. Y. X. Benbasat I. (2008). A two-process view of trust and distrust building in recommendation agents: a process-tracing study. J. Assoc. Inf. Syst. 9, 727–747. doi: 10.17705/1jais.00180

[B25] Liang F. Paulo R. Molina G. Clyde M. A. Berger J. O. (2008). Mixtures of g priors for Bayesian variable selection. J. Am. Stat. Assoc. 103, 410–423. doi: 10.1198/016214507000001337

[B26] Longoni C. Bonezzi A. Morewedge C. K. (2019). Resistance to medical artificial intelligence. J. Consum. Res. 46, 629–650. doi: 10.1093/jcr/ucz013

[B27] Malhotra N. K. Kim S. S. Agarwal J. (2004). Internet users' information privacy concerns (IUIPC): The construct, the scale, and a causal model. Inf. Syst. Res. 15, 336–355. doi: 10.1287/isre.1040.0032

[B28] Mao K. Cui R. Wang Z. (2026). Contextualizing the privacy paradox–a risk-benefit analysis of generation z's adoption intentions toward AI-based virtual try-on. Front. Psychol. 17:1773754. doi: 10.3389/fpsyg.2026.1773754 42039070 PMC13106007

[B29] Martin K. D. Murphy P. E. (2017). The role of data privacy in marketing. J. Acad. Market. Sci. 45, 135–155. doi: 10.1007/s11747-016-0495-4

[B30] McKnight D. H. Choudhury V. Kacmar C. (2002). Developing and validating trust measures for e-commerce: an integrative typology. Inf. Syst. Res. 13, 334–359. doi: 10.1287/isre.13.3.334.81

[B31] Mekioussa Malki L. (2026). “Towards usable, privacy respecting long-term memory for llm-based conversational agents,” in Proceedings of the extended abstracts of the 2026 CHI conference on human factors in computing systems (New York, NY: ACM), 1–5. doi: 10.1145/3772363.3799198

[B32] Nissenbaum H. (2010). Privacy in Context: Technology, Policy, and the Integrity of Social Life. Stanford, CA: Stanford University Press. doi: 10.1515/9780804772891

[B33] Norberg P. A. Horne D. R. Horne D. A. (2007). The privacy paradox: personal information disclosure intentions versus behaviors. J. Consum. Aff. 41, 100–126. doi: 10.1111/j.1745-6606.2006.00070.x

[B34] Pavlou P. A. (2003). Consumer acceptance of electronic commerce: integrating trust and risk with the technology acceptance model. Int. J. Electron. Commer. 7, 101–134. doi: 10.1080/10864415.2003.11044275

[B35] Petronio S. (2002). Boundaries of Privacy: Dialectics of Disclosure. Albany, NY: State University of New York Press. doi: 10.1353/book4588

[B36] Petronio S. (2013). Brief status report on communication privacy management theory. J. Fam. Commun. 13, 6–14. doi: 10.1080/15267431.2013.743426

[B37] Petrova A. Malär, L. Hoyer W. Krohmer H. (2026). The phenomenon of creepiness in a digital marketing world. Psychol. Market. 43, 834–851. doi: 10.1002/mar.70089

[B38] Puntoni S. Reczek R. W. Giesler M. Botti S. (2021). Consumers and artificial intelligence: an experiential perspective. J. Mark. 85, 131–151. doi: 10.1177/0022242920953847

[B39] Rouder J. N. Morey R. D. Speckman P. L. Province J. M. (2012). Default Bayes factors for ANOVA designs. J. Math. Psychol. 56, 356–374. doi: 10.1016/j.jmp.2012.08.001

[B40] Smith H. J. Milberg S. J. Burke S. J. (1996). Information privacy: measuring individuals' concerns about organizational practices. MIS Q. 20, 167–196. doi: 10.2307/249477

[B41] Tene O. Polonetsky J. (2014). A theory of creepy: technology, privacy, and shifting social norms. Yale J. Law Technol. 16, 59–102. doi: 10.2139/ssrn.2326830

[B42] Tucker C. E. (2014). Social networks, personalized advertising, and privacy controls. J. Mark. Res. 51, 546–562. doi: 10.1509/jmr.10.0355

[B43] Wagenmakers E.-J. (2007). A practical solution to the pervasive problems of p values. Psychon. Bullet. Rev. 14, 779–804. doi: 10.3758/BF03194105 18087943

[B44] Wang W. Benbasat I. (2005). Trust in and adoption of online recommendation agents. J. Assoc. Inf. Syst. 6, 72–101. doi: 10.17705/1jais.00065

[B45] Xu H. Luo X. R. Carroll J. M. Rosson M. B. (2011). The personalization privacy paradox: an exploratory study of decision making process for location-aware marketing. Decis. Support Syst. 51, 42–52. doi: 10.1016/j.dss.2010.11.017

[B46] Yeomans M. Shah A. Mullainathan S. Kleinberg J. (2019). Making sense of recommendations. J. Behav. Decis. Mak. 32, 403–414. doi: 10.1002/bdm.2118

[B47] Zhang S. Ma R. Ma Y. Li S. Xu Y. Yi X. . (2025). “Understanding users' privacy perceptions towards LLM's RAG-based memory,” in Proceedings of the 2025 workshop on human-centered AI privacy and security (New York, NY: ACM), 10–19. doi: 10.1145/3733816.3760750

[B48] Zhu L. Zhang C. Omar B. Qi F. Ji H. (2025). Privacy threats versus trust: a behavioral decision approach to social media disclosure intention. Front. Psychol. 16:1609012. doi: 10.3389/fpsyg.2025.1609012 41079871 PMC12510261

