# Supplementary material for: Remembering or monitoring? Contextual fit of simulated AI memory cues and consumer trust
Source: Front Psychol. 2026 Sep 10;17:1934857. doi: 10.3389/fpsyg.2026.1934857 (PMC13600881; doi:10.3389/fpsyg.2026.1934857)
Supplement: Supplementary file 1 [file Supplementary_File_1.pdf]

Supplementary Materials

Manuscript: *Remembering or monitoring? Contextual fit of simulated AI memory cues and consumer trust*

Supplementary Table S1. Sample and data-quality flow

Participants were recruited as a mixed sample through Zhejiang Business College course-participation channels (students, compensated with course credit) and on-campus community recruitment events (local adult residents from the surrounding community, compensated with small gifts). Before analysis, responses were screened against four exclusion criteria: failed attention or comprehension checks; data-entry errors (incomplete, malformed, or duplicated submissions); insufficient completion time (less than one-third of the study median); and erroneous content (stimulus-delivery compliance failures or off-task free-text input). Across the six studies, 2,302 responses were screened and 162 (7.0%) were excluded, leaving 2,140 in the final analytic samples. The de-identified analysis files contain the final valid responses after screening. Raw screening logs and demographic records are retained separately from the public analysis files to protect participant privacy.

Study file	Screened	Excluded	Final analytic N
`study1_memory_data.csv`	286	22	264
`study2_memory_data.csv`	307	18	289
`study3_memory_data.csv`	441	31	410
`study4_memory_data.csv`	557	41	516
`study5_memory_data.csv`	342	24	318
`study6_memory_data.csv`	369	26	343
**Total**	**2,302**	**162**	**2,140**

Exclusions by reason. The screening logs record the reason for every excluded response. Reasons are reported per study; because the raw screening logs are stored separately from the de-identified analysis files under the IRB protocol, they cannot be cross-tabulated by experimental condition.

Study	Failed attention check	Data-entry error	Insufficient duration	Erroneous content	Total excluded
1	8	6	5	3	22
2	6	4	5	3	18
3	11	7	8	5	31
4	14	9	11	7	41
5	8	5	7	4	24
6	9	6	7	4	26
**Total**	**56**	**37**	**43**	**26**	**162**

Retained condition cell sizes. The experiment program assigned conditions in balanced blocks, and responses excluded by the screening rules were replaced by continued recruitment within the same window, so the retained cells remain balanced (equal across the second factor in the factorial designs of Studies 3 and 4). Recruitment was never conditioned on the outcome data; no interim analyses were conducted. Raw screening logs are stored separately from the de-identified files under the IRB protocol, so exclusions are reported per study and per reason, as above, but cannot be cross-tabulated by condition. The retained counts show no obvious gross depletion of a single condition, but they do not permit a direct test of condition-specific exclusion rates:

Study	Condition cells (retained n)
1	No Memory 91 / Relevant 88 / Intrusive 85
2	No Memory 96 / Relevant 99 / Intrusive 94
3	Six cells (Memory Type × Scenario): 70/70 (No), 67/67 (Relevant), 68/68 (Intrusive) for apparel/sleep-health scenarios
4	Six cells (Memory Type × Specific dashboard): 88/88 (No), 84/84 (Relevant), 86/86 (Intrusive) for dashboard present/absent
5	No Memory 108 / Relevant 106 / Intrusive 104
6	No Memory 116 / Relevant 115 / Intrusive 112

Variables in each de-identified analysis file:

Study file	Variables
`study1_memory_data.csv`	Participant_ID, Age, Gender, Grade, Testing_Channel, Memory_Type, Memory_Code, MBF, Trust_Item1-3, Purchase_Item1-3, Continue_Item1-3
`study2_memory_data.csv`	Participant_ID, Age, Gender, Grade, Testing_Channel, Memory_Type, Memory_Code, MBF, Relational_Warmth, Surveillance_Concern, Trust_Item1-3
`study3_memory_data.csv`	Participant_ID, Age, Gender, Grade, Testing_Channel, Sensitivity, Memory_Type, Trust_Item1-3
`study4_memory_data.csv`	Participant_ID, Age, Gender, Grade, Testing_Channel, Memory_Type, Control_Interface, MBF, Trust_Item1-3
`study5_memory_data.csv`	Participant_ID, Age, Gender, Grade, Testing_Channel, Memory_Type, MBF, Trans_Item1-3, DataShare_Item1-3, Rel_Item1-3

Study file	Variables
`study6_memory_data.csv`	Participant_ID, Age, Gender, Grade, Testing_Channel, Memory_Type, Memory_Code, MBF, PrivacyTrust_Item1-3, Loyalty_Item1-3, OptIn_Item1-3, PlatformTrust_Item1-3

Note. `Sensitivity` and `Control_Interface` are legacy field names retained so that the data dictionary remains aligned with the archived analysis files. In the manuscript, `Sensitivity` is interpreted only as the apparel-versus-sleep-health scenario indicator, and `Control_Interface` denotes the specific inspect-edit-delete dashboard tested.

Supplementary Table S2. Sample composition and demographics

The participant pool was a mixed sample of Zhejiang Business College students and local adult residents from the surrounding community. Students were recruited through campus-based course participation, and residents through on-campus community recruitment events; both groups could also participate through online/network testing links using the same survey materials. `Grade=Resident` denotes community adult residents; `Year1--3` denotes enrolled students by class year.

Study	N	Age M (SD)	Female	Male	Prefer not say	Year 1	Year 2	Year 3	Resident	Campus session	Campus resident session	Online/ network
Overall	2140	26.18 (10.61)	58.3%	40.1%	1.6%	22.9%	25.3%	23.2%	28.6%	42.7%	28.6%	28.8%
`study1_memory_data.csv`	264	24.39 (9.08)	55.3%	43.2%	1.5%	27.3%	27.7%	23.5%	21.6%	46.2%	21.6%	32.2%
`study2_memory_data.csv`	289	25.35 (10.13)	58.8%	39.8%	1.4%	18.7%	30.1%	27.0%	24.2%	48.8%	24.2%	27.0%
`study3_memory_data.csv`	410	24.79 (9.98)	58.5%	39.5%	2.0%	26.3%	28.8%	23.4%	21.5%	44.6%	21.5%	33.9%
`study4_memory_data.csv`	516	28.47 (11.66)	57.2%	40.9%	1.9%	20.5%	20.5%	20.2%	38.8%	39.3%	38.8%	21.9%
`study5_memory_data.csv`	318	25.27 (10.08)	59.7%	38.1%	2.2%	23.9%	27.4%	24.8%	23.9%	44.7%	23.9%	31.4%
`study6_memory_data.csv`	343	27.33 (11.01)	60.1%	39.7%	0.3%	21.9%	20.4%	22.7%	35.0%	35.6%	35.0%	29.4%

Supplementary Table S3. Manipulation checks

In every study that measured it, MBF differed by memory condition in the theoretically expected direction, indicating that the complete cue packages changed the measured contextual-appropriateness appraisal. In each study, MBF was highest under relevant memory and lowest under intrusive memory, with the no-memory condition scoring between them, and the omnibus effect of memory type was significant (all $p < .001$). Because no remembered content is present in the no-memory condition, its score is interpreted as a neutral reference point rather than as an intermediate level of fit. Because MBF served as both the manipulation check and the intermediate variable in the indirect-association models, these checks do not independently establish a psychological mechanism. The explanatory analyses therefore give primary weight to the randomized relevant-versus-intrusive cue-package contrast.

Study	Ordering of MBF by condition
Study 1	Relevant > No memory > Intrusive
Study 2	Relevant > No memory > Intrusive
Study 4	Relevant > No memory > Intrusive
Study 5	Relevant > No memory > Intrusive
Study 6	Relevant > No memory > Intrusive

All omnibus tests were significant at $p < .001$.

Scale items and measurement documentation

Complete measurement documentation is provided in Supplementary Note S14, which reports the administration format, response anchors, scoring rules, presentation order, the source scale from which each construct was adapted, the administered wording with verified back-translation for every measure, and item-level psychometric evidence for the downstream scales.

Stimulus summary

- **No memory**: The assistant provided a generic response without referencing previous interactions.
- **Relevant memory**: The assistant referenced task-relevant prior preferences such as budget, fit, comfort, or product features.
- **Intrusive memory**: The assistant referenced sensitive, cross-contextual, or excessive information such as late-night browsing, stress cues, or unrelated prior behaviour.
- **Editable dashboard**: In Study 4, the interface displayed controls to inspect, edit, or delete stored memories.

The full system prompt, few-shot constraints, decoding settings, per-condition example exchanges, and the output quality-control procedure are documented in `stimulus_protocol.md`. Within each scenario and dashboard level, memory-related wording appeared inline in the assistant's chat message with identical formatting and visual styling across memory-type conditions. The core recommendation set remained fixed within each scenario. Figure 1 of the manuscript is a schematic for readers, not a screenshot of the participant-facing interface.

Supplementary Table S4. Reproducibility materials

The analysis scripts (`full_analysis.py`, `bootstrap_indirect_effects.py`, `bayes_null_tests.py`, `revision_analyses.py`, `generate_nature_figures.py`, `generate_figure1_schematic.py`, `mbf_validation_analysis.py`), cleaned data, stimulus protocol, and exact statistics files (`exact_stats.json`, `bayes_null_tests.json`, `revision_analyses.json`) are retained with the project materials to support reproducibility.

`revision_analyses.py` reproduces every analysis added during revision from the public de-identified data files: the Holm–Bonferroni multiplicity control and the hypothesis-specific DiD contrasts, the HC3-robust re-estimation of the Study 3 focal tests, the standardized pairwise effect sizes, the testing-channel robustness checks and ANOVA assumption diagnostics (Supplementary Note S13); the explanatory models restricted to the relevant-versus-intrusive subsample (Supplementary Table S9b); the item-level psychometrics for the downstream scales (Supplementary Note S14.5); and the bootstrap comparison of dependent correlations sharing MBF (Supplementary Table S11). It writes `analysis_outputs/revision_analyses.json` and uses a fixed seed (20260722) for all bootstrap procedures.

The analyses used Python 3 with pandas, NumPy, SciPy, and statsmodels. The repository-level `requirements.txt` records minimum compatible versions (`pandas>=2.0`, `numpy>=1.24`, `scipy>=1.10`, and `statsmodels>=0.14`). The archived project does not contain a historical lockfile or environment export, so exact package patch versions used for the original runs cannot be verified and are not reconstructed here.

Supplementary Table S5. MBF discriminant-validity checks

Table S5a. Study 2 inter-construct correlations

Study 2 ($N = 289$) measured MBF alongside relational warmth and surveillance concern to probe whether MBF is distinct from adjacent evaluations. Correlations are moderate rather than near-unity: MBF relates positively to warmth and trust and negatively to surveillance concern, but is not redundant with any of them.

Construct	1	2	3	4
1. MBF	—			
2. Relational warmth	.42	—		
3. Surveillance concern	-.55	-.33	—	
4. Trust	.57	.25	-.32	(.88)

Note. MBF, relational warmth, and surveillance concern are single composite scores in the Study 2 public analysis file; the value in parentheses on the diagonal is the Cronbach's α for the multi-item trust scale. Correlations computed from Study 2.

Table S5b. Supplemental item-level MBF validation sample

A supplemental validation sample ($N = 234$) completed the three MBF items together with IUIPC privacy concern (9 items), relational warmth (3 items), surveillance concern (3 items), and trust (3

items). This validation dataset is stored separately in the `validation/` folder and is not included in the six-study experimental N of 2,140. It was used to evaluate the three-item MBF scale at the item level because the six focal experiment files retain MBF as a composite score.

Check	Result	Interpretation
MBF standardized loadings	.623, .882, .659 (closed form); .582, .912, .657 (five-factor ML CFA)	Uneven; one indicator carries most of the common variance
MBF Cronbach's α	.761	Acceptable internal consistency
MBF composite reliability (CR)	.770 (.768 from the CFA loadings)	Above .70 criterion
MBF average variance extracted (AVE)	.533 (.534 from the CFA loadings)	Above .50 criterion, but by a modest margin
Five-factor CFA	$\chi^2(179) = 197.85$; CFI = .990; RMSEA = .021; SRMR = .045	Good approximate fit
Fornell–Larcker	$\sqrt{\text{AVE}} = .730$; max |MBF composite correlation| = .649 (trust)	Passed, narrow margin
Max MBF HTMT	.815 (MBF–trust)	Below .85 criterion, but close to it

Note. CR and AVE are computed from a common-factor solution. Because the one-factor model for three indicators is just-identified, the standardized loadings follow in closed form from the inter-item correlations, and the maximum-likelihood five-factor CFA reproduces them to within .04. A principal-component (first-component) solution would absorb unique as well as common variance and would inflate both indices for a scale this short; it is not used here.

MBF composite correlations in the validation sample were moderate with IUIPC privacy concern ($r = -.408$), relational warmth ($r = .439$), surveillance concern ($r = -.470$), and trust ($r = .649$). HTMT ratios were .492 for IUIPC, .599 for warmth, .599 for surveillance concern, and .815 for trust. These results support the three-item MBF scale as an initial operationalization that is related to, but separable from, adjacent privacy, surveillance, warmth, and trust constructs. The separation from trust is the weakest of the four, and it deserves to be stated plainly. It satisfies both the Fornell–Larcker criterion ($\sqrt{\text{AVE}} = .730$ for MBF and .776 for trust, against $r = .649$) and the HTMT criterion ($.815 < .85$), but with little room to spare under either. Correcting the composite correlation for attenuation using the two composite reliabilities gives an estimated true-score correlation of .82, so the two constructs share roughly two thirds of their reliable variance in this sample. They are separable by the conventional criteria, but they are not far apart, and the incremental-validity evidence in Note S15 is subject to the common-method concern noted there. A reader should therefore treat MBF as a distinguishable but closely related antecedent of trust rather than as a clearly independent construct. Refinement of the item set, in particular of the two weaker indicators, is a priority for further work on the scale.

Supplementary Table S6. Sensitivity to participant status (student vs. community resident)

Because the samples mixed enrolled students (`Year1--3`) with community adult residents (`Resident`, 28.6% overall), we re-estimated every focal effect with participant status added as a covariate and tested whether memory type interacted with status. Across all focal outcomes the significance judgment was unchanged when status was controlled, and no Memory Type × Status interaction reached significance (all *p*s > .13). Status itself did not produce a significant main effect on any outcome (all *p*s > .05), indicating that the mixed sample did not introduce systematic mean-level differences. The selected Study 2 trust and Study 6 loyalty indirect-association paths displayed in Table S6c were also re-estimated within each subgroup and remained consistent in sign and order of magnitude. Together these results indicate that the memory-type effects reported in the main text are robust to sample composition; they do not broaden generalization beyond the sampled population.

Table S6a. Focal effects with participant status as a covariate.

Study	Outcome	Orig. F	Orig. p	Orig. η_p^2	+Status F	+Status p	+Status η_p^2
Study 1	Trust	5.96	.003	.044	6.04	.003	.044
Study 1	Purchase intention	1.76	.175	.013	1.64	.196	.012
Study 1	Continued use	3.98	.020	.030	3.90	.021	.029
Study 2	Trust	19.86	<.001	.122	19.71	<.001	.122
Study 3	Memory × Scenario	4.82	.009	.023	4.62	.010	.022
Study 4	Memory × Specific dashboard	0.54	.584	.002	0.51	.600	.002
Study 5	Data-sharing engagement	24.03	<.001	.132	23.94	<.001	.132
Study 5	Transactional engagement	1.95	.144	.012	2.01	.136	.013
Study 5	Relational engagement	14.80	<.001	.086	14.84	<.001	.086
Study 6	Loyalty	9.77	<.001	.054	9.90	<.001	.055
Study 6	Opt-in intention	17.13	<.001	.092	17.10	<.001	.092

Note. For Studies 1, 2, 5, and 6 the focal effect is the memory-type main effect; for Studies 3 and 4 it is the focal two-way interaction. +Status models add `Status` (student vs. resident) as a covariate to the original model.

Table S6b. Memory Type × Status interaction tests.

Study	Outcome	Memory Type × Status F	p
Study 1	Trust	0.60	.550
Study 1	Purchase intention	0.57	.566
Study 1	Continued use	0.52	.592
Study 2	Trust	1.58	.208
Study 5	Data-sharing engagement	0.60	.550
Study 5	Transactional engagement	1.99	.139
Study 5	Relational engagement	1.76	.173
Study 6	Loyalty	0.96	.385
Study 6	Opt-in intention	0.99	.371

Note. None of the interactions reached significance (all ps $>$ .13), providing no evidence that the memory-type effect differed between students and community residents.

Table S6c. Indirect associations within each subgroup.

Study	Path	Student n	Student indirect	Resident n	Resident indirect
Study 2	Memory → MBF → Trust	219	0.36	70	0.48
Study 6	Memory → MBF → Privacy trust → Loyalty (serial)	223	0.15	120	0.14

Note. The two displayed paths retain the same sign and a comparable order of magnitude within each subgroup, consistent with the corresponding overall indirect-association estimates reported in the main text. Table S6c does not report every downstream Study 6 outcome. These same-session subgroup models do not establish causal ordering.

Supplementary Table S7. Competitive indirect-association model (Study 2)

Because MBF, relational warmth, and surveillance concern are conceptually adjacent evaluations, we tested whether MBF retains a reliable indirect association once the two competing appraisals are included. In the conventional multiple-mediator specification, the *specific* indirect association through each appraisal is the product of the effect of memory type on that appraisal (a) and the association of that appraisal with trust controlling for the other appraisals and memory code (b). Specific indirect associations were evaluated with 5,000 percentile bootstrap resamples (fixed seed; `code/bootstrap_indirect_effects.py`). Memory type enters this model with the ordinal code used for the summary models in Table S9 (1 = intrusive, 2 = no memory, 3 = relevant), so that each appraisal has a single a path and the three competitors are directly comparable. Because the main text does not treat ordinal coding as primary, we re-estimated the model two further ways; the conclusion is unchanged under both (see the robustness rows below). Because all appraisals and trust were measured in the same session, this model does not establish causal transmission or temporal ordering.

Intermediate appraisal	a	b	Specific indirect association	95% CI
MBF	0.80	0.47	0.38	[0.27, 0.50]
Relational warmth	0.67	-0.01	-0.01	[-0.09, 0.07]
Surveillance concern	-0.91	0.02	-0.02	[-0.12, 0.10]

Robustness to the coding of memory type. Re-estimating the same three-appraisal model with dummy codes (no memory as reference) and on the relevant-versus-intrusive subsample gives the same conclusion: only MBF has a specific indirect association whose interval excludes zero.

Coding	MBF	Relational warmth	Surveillance concern
Ordinal (as above)	0.383 [0.275, 0.504]	−0.008 [−0.092, 0.070]	−0.017 [−0.123, 0.097]
Dummy: relevant vs. no memory	0.335 [0.182, 0.505]	−0.008 [−0.092, 0.074]	−0.017 [−0.115, 0.087]
Dummy: intrusive vs. no memory	−0.429 [−0.610, −0.270]	0.008 [−0.070, 0.096]	0.021 [−0.112, 0.141]
Relevant vs. intrusive subsample ($n = 193$)	0.725 [0.468, 0.991]	−0.016 [−0.245, 0.202]	−0.031 [−0.292, 0.248]

Pairwise contrasts between the specific indirect associations. A difference in whether two confidence intervals exclude zero is not itself evidence that the two associations differ. We therefore bootstrapped the pairwise contrasts directly, on the same 5,000 resamples used above.

Contrast	Difference	95% CI
MBF − relational warmth	0.391	[0.244, 0.546]
MBF − surveillance concern	0.400	[0.233, 0.576]
Relational warmth − surveillance concern	0.009	[−0.136, 0.146]

Note. b is the coefficient on the focal appraisal with all three appraisals and memory code entered jointly. MBF is the only appraisal whose specific indirect association has a 95% confidence interval excluding zero, and the direct contrasts confirm that its specific indirect association is larger than that of either competitor, while the two competitors do not differ from each other. This indicates that MBF is not statistically interchangeable with warmth or surveillance concern in this model. It does not establish MBF as the causal mechanism: all three appraisals and trust were measured in the same session, the contrast is between measured covariances rather than manipulated attributes, and the model does not test usefulness, intrusiveness, creepiness, information relevance, or perceived norm violation, none of which were measured.

Supplementary Table S8. Tukey HSD pairwise comparisons for all focal outcomes

Pairwise mean differences with Tukey-adjusted 95% confidence intervals and p-values, computed from the de-identified analysis files. Positive differences indicate that the first-named condition scored

higher. R = Relevant Memory, N = No Memory, I = Intrusive Memory.

Study	Outcome	R − N (benefit)	I − N (negative indicates penalty)	R − I
1	Trust	0.40 [0.03, 0.76], $p = .028$	-0.12 [-0.48, 0.25], $p = .736$	0.51 [0.14, 0.89], $p = .003$
1	Purchase intention	0.14 [-0.21, 0.49], $p = .601$	-0.14 [-0.49, 0.21], $p = .619$	0.28 [-0.07, 0.63], $p = .148$
1	Continued use	0.36 [0.01, 0.70], $p = .040$	-0.01 [-0.35, 0.34], $p = .999$	0.36 [0.01, 0.71], $p = .040$
2	Trust	0.31 [-0.03, 0.66], $p = .076$	-0.59 [-0.93, -0.24], $p < .001$	0.90 [0.56, 1.25], $p < .001$
3 (apparel)	Trust	0.51 [0.10, 0.92], $p = .010$	-0.4099 [-0.8202, 0.0004], $p = .0503$	0.92 [0.51, 1.34], $p < .001$
3 (sleep health)	Trust	0.73 [0.30, 1.16], $p < .001$	-0.95 [-1.38, -0.53], $p < .001$	1.68 [1.25, 2.11], $p < .001$
4 (pooled)	Trust	0.37 [0.14, 0.59], $p < .001$	-0.47 [-0.70, -0.24], $p < .001$	0.83 [0.61, 1.06], $p < .001$
5	Transactional eng.	0.12 [-0.21, 0.46], $p = .668$	-0.16 [-0.50, 0.18], $p = .499$	0.28 [-0.06, 0.62], $p = .121$
5	Data-sharing eng.	0.66 [0.34, 0.99], $p < .001$	-0.27 [-0.60, 0.05], $p = .118$	0.94 [0.61, 1.26], $p < .001$
5	Relational eng.	0.40 [0.08, 0.72], $p = .010$	-0.34 [-0.66, -0.02], $p = .032$	0.74 [0.42, 1.06], $p < .001$
6	Loyalty	0.29 [-0.01, 0.59], $p = .055$	-0.27 [-0.57, 0.03], $p = .085$	0.56 [0.26, 0.86], $p < .001$
6	Opt-in intention	0.31 [0.01, 0.61], $p = .038$	-0.43 [-0.74, -0.13], $p = .002$	0.75 [0.45, 1.05], $p < .001$

Note. The relevant-versus-intrusive contrast (R − I) is the principal and most consistent comparison: it is significant for every outcome except Study 1 purchase intention and Study 5 transactional engagement (the two outcomes whose omnibus tests were also null). Its two comparisons with no memory dissociate across studies. The benefit component (R − N) is reliable for Study 1 trust and continued use, both Study 3 scenarios, Study 4 trust, Study 5 data-sharing and relational engagement, and Study 6 opt-in intention. The penalty component is defined as N − I; equivalently, it appears as a reliable negative I − N difference in the table for Study 2 trust, the Study 3 sleep-health scenario, Study 4 trust, Study 5 relational engagement, and Study 6 opt-in intention. Both components are reliable simultaneously for the Study 3 sleep-health scenario, Study 4 trust, Study 5 relational engagement, and Study 6 opt-in intention. The benefit and penalty should therefore not be regarded as uniformly established relative to no memory.

Supplementary Table S9. Relative indirect associations with dummy-coded memory type

Because the three-level memory factor is categorical, the Study 2 and Study 6 indirect-association models were estimated with two dummy codes using no memory as the reference category, yielding relative indirect associations (Hayes & Preacher, 2014). Confidence intervals are 95% percentile bootstrap intervals (5,000 resamples, fixed seed). The models are conventionally described as mediation and serial-mediation models, but the same-session mediator-to-outcome paths are interpreted here as associations rather than causal mechanisms.

Study 2: memory type → MBF → trust. Path estimates: a(relevant) = 0.71 ($p < .001$), a(intrusive) = -0.91 ($p < .001$), b(MBF) = 0.47 ($p < .001$); relative direct effects: relevant −0.01 ($p = .910$), intrusive −0.17 ($p = .220$).

Contrast	Relative indirect association	95% CI
Relevant vs. no memory	0.33	[0.18, 0.51]
Intrusive vs. no memory	-0.42	[-0.60, -0.27]

Study 6: memory type → MBF → privacy trust → loyalty (serial).

Contrast	Relative serial indirect association	95% CI
Relevant vs. no memory	0.14	[0.08, 0.21]
Intrusive vs. no memory	-0.15	[-0.23, -0.09]

Study 6: memory type → MBF → privacy trust → opt-in intention (serial).

Contrast	Relative serial indirect association	95% CI
Relevant vs. no memory	0.14	[0.08, 0.21]
Intrusive vs. no memory	-0.15	[-0.23, -0.09]

Note. These dummy-coded models are the primary indirect-association analyses reported in the main text. All six relative indirect associations exclude zero and are of opposite sign for the two contrasts. The ordinal-coded summaries converge with them, indicating that the numerical pattern does not depend on imposing a metric on the memory factor. This convergence does not resolve the causal-ordering limitation. The ordinal models are retained only as summaries because the no-memory condition presents no remembered content to evaluate and its MBF score is not interpreted as an intermediate level of fit.

Ordinal-coded summary models (1 = intrusive, 2 = no memory, 3 = relevant). Study 2: indirect association 0.38, 95% CI [0.28, 0.49]; direct association $p = .327$. Study 6: serial indirect association 0.15 for loyalty intention and 0.15 for opt-in intention, 95% CIs [0.10, 0.20].

Supplementary Table S10. Achieved-sample power bounds for the experimental test family

The table reports the minimum effect size detectable with 80% power at $\alpha = .05$ for each study's experimental test in the original six-study multiplicity family (numerator $df = 2$ throughout), com-

puted from the noncentral F distribution. These power bounds do not apply to the heterogeneous hypothesis-specific association tests summarized in Table 2 of the main text.

Study	Experimental test	N	Min. detectable f	Min. detectable η_p^2
1	Memory type (one-way, $k = 3$)	264	0.192	.036
2	Memory type (one-way, $k = 3$)	289	0.184	.033
3	Memory × Scenario interaction	410	0.154	.023
4	Memory × Specific dashboard interaction	516	0.137	.018
5	Memory type (one-way, $k = 3$)	318	0.175	.030
6	Memory type (one-way, $k = 3$)	343	0.168	.028

Note. Two reported null results can be read against these same-design bounds. Study 1's purchase-intention effect ($\eta_p^2 = .013$) falls below that study's detectable range (.036). Study 4's Memory × Specific Dashboard interaction ($\eta_p^2 = .002$) is far below the detectable bound (.018), aligning with the main-text interpretation that this particular dashboard implementation produced a general trust benefit rather than selective attenuation of the intrusive-memory penalty. This is not evidence about user control generally. Because both effects fall below what their designs could detect, these nulls are uninformative rather than evidence of absence, and they should be read together with the prior-sensitivity analysis in Table S12: the BIC approximation reported there would describe them as strong evidence for the null, but the JZS default does not, and we adopt the latter reading.

Supplementary Table S11. Study 5 MBF-outcome associations

Because H5 concerns the relationship between MBF and engagement type, we supplemented the memory-type ANOVAs with direct MBF-outcome correlations in Study 5 and bootstrap comparisons of dependent correlations sharing the same MBF variable (5,000 resamples, fixed seed).

Outcome	Correlation with MBF	p
Data-sharing engagement	.303	$< .001$
Transactional engagement	-.045	.423
Relational engagement	.195	$< .001$

Correlation contrast	Difference in r	95% CI
MBF–data-sharing minus MBF–transactional	.348	[.20, .49]
MBF–data-sharing minus MBF–relational	.108	[-.03, .24]
MBF–relational minus MBF–transactional	.240	[.08, .40]

Note. The theoretically focal contrast is data-sharing versus transactional engagement. Data-sharing engagement was more strongly associated with MBF than transactional engagement; its difference from relational engagement was directionally positive but not statistically reliable.

Supplementary Table S12. Bayes factors for focal null results under two default priors

The strength of evidence for a null hypothesis depends on the prior placed on the alternative, and the two conventional defaults diverge sharply in designs of this size. We therefore report both, and base our interpretation on the more conservative of the two.

The first is the BIC approximation (Wagenmakers, 2007), comparing the no-effect (or additive) model against the model containing the focal term:

$$\mathrm{BF}_{01} = \exp\left\{\tfrac{1}{2}\left(\mathrm{BIC}_{\mathrm{alt}} - \mathrm{BIC}_{\mathrm{null}}\right)\right\}.$$

This approximation corresponds to a unit-information prior, that is, a g-prior with $g = n$. Because that prior is very diffuse, it imposes a large Ockham penalty on the alternative and is strongly biased toward the null at these sample sizes. The second is the Jeffreys–Zellner–Siow (JZS) mixture-of-g-priors Bayes factor computed from the same nested F test (Liang et al., 2008), which places a Cauchy prior on the standardized effect and belongs to the same family as the defaults implemented in common Bayesian ANOVA software (Rouder et al., 2012). Values below are reported at the unit Cauchy scale ($r = 1$).

Study	Focal term	DV	F (p)	BF_{01} BIC	BF_{01} JZS	Interpretation adopted
1	Memory type (omnibus)	Purchase intention	$F(2, 261) = 1.76$ ($p = .175$)	45.2	1.22	Undetermined; effect below the detectable bound
4	Memory × Specific dashboard int.	Trust	$F(2, 510) = 0.54$ ($p = .584$)	299.5	2.26	Consistent with the additive model; not decisive
4	Memory × Specific dashboard int.	MBF	$F(2, 510) = 1.74$ ($p = .177$)	89.4	1.23	Undetermined
4	Specific dashboard main effect	MBF	$F(1, 510) = 3.77$ ($p = .053$)	3.45	0.65	Priors disagree in direction; undetermined. Descriptive direction (dashboard lower) is opposite to H4 and is not interpreted
5	Memory type (omnibus)	Transactional eng.	$F(2, 315) = 1.95$ ($p = .144$)	44.9	1.10	Undetermined

Sensitivity to the Cauchy prior scale. Because a Bayes factor for a null depends on the prior scale as well as the prior family, we recomputed each comparison across a range of scales. The BIC value is shown for reference.

Test	BIC	$r = 0.5$	$r = 0.707$	$r = 1$	$r = 1.414$	$r = 2$
S4 Memory × dashboard (trust)	299.5	1.53	1.81	2.26	3.03	4.39
S4 Memory × dashboard (MBF)	89.4	1.04	1.11	1.23	1.46	1.87
S4 Dashboard main effect (MBF)	3.45	0.69	0.66	0.65	0.65	0.70
S5 Memory type (transactional)	44.9	0.97	1.01	1.10	1.27	1.60
S1 Memory type (purchase)	45.2	1.04	1.10	1.22	1.44	1.85

Across the whole range the largest value is $BF_{01} = 4.39$, and only one comparison exceeds 3 at any scale. The gap between the BIC approximation and this family is therefore a property of the unit-information prior, not an artefact of one particular Cauchy scale.

Note. JZS Bayes factors are computed from the nested F test for the focal term; for the Study 4 dashboard main effect the nested comparison is against the additive model, giving $F(1, 512) = 3.76$, which differs trivially from the full-factorial $F(1, 510) = 3.77$ reported in the main text. Under the BIC approximation four of the five comparisons would be described as strong evidence for the null. Under the JZS prior none of them exceeds $BF_{01} = 3$ at the unit scale, and one falls below 1. This pattern is what the achieved-sample power bounds in Table S10 would lead one to expect: these designs could not detect effects of the observed size, so the null results are uninformative rather than positive evidence of absence. We therefore describe them throughout as consistent with no effect of the size each design could detect, and we do not claim evidence that any of these effects is absent. This applies in particular to the Study 4 interaction, which bears on H4.

Script: `code/bayes_null_tests.py`; output archived in `analysis_outputs/bayes_null_tests.json`.

Supplementary Note S13. Multiplicity control, expectation-aligned contrasts, and testing-channel robustness (added in revision)

Multiplicity across the original six-study experimental test family. A Holm–Bonferroni adjustment was applied to the original family containing one experimental omnibus or interaction p value per study, listed below. This family is distinct from the heterogeneous hypothesis-specific results summarized in Table 2 of the main text; the adjustment is not presented as covering every coefficient or indirect association. All experimental effects in this family that were significant without adjustment remained significant after adjustment; the Study 4 interaction remained nonsignificant. Although the Study 3 comparison was motivated by an a priori sensitivity expectation, it is interpreted as an exploratory comparison of two complete scenario packages because sensitivity was not isolated.

Experimental test	Unadjusted p	Holm-adjusted p	Significant after adjustment
Study 1: memory type → trust	.0029	.0088	Yes
Study 2: memory type → trust (omnibus)	8.4e-09	4.2e-08	Yes
Study 3: Memory × Scenario interaction	.0086	.0171	Yes; exploratory package comparison
Study 4: Memory × Specific dashboard interaction	.584	.584	No
Study 5: memory type → data-sharing eng.	1.9e-10	1.2e-09	Yes
Study 6: memory type → opt-in intention	8.1e-08	3.3e-07	Yes

Difference-in-differences contrasts corresponding to the original H3 and H4 expectations. The reported contrast is the change in the intrusive-memory penalty (no memory minus intrusive memory) across the two scenarios in Study 3 or the two dashboard levels in Study 4. Contrasts were estimated as linear combinations of cell means in the cell-means OLS model. Because Study 3 bundled several scenario characteristics, its contrast remains an exploratory scenario-package comparison rather than a direct test of sensitivity.

Study	Contrast	Estimate	SE	t (df)	p	95% CI
3	Penalty(sleep-health scenario) − Penalty(apparel scenario)	0.54	0.25	2.17 (404)	.031	[0.05, 1.04]
4	Penalty(dashboard present) − Penalty(dashboard absent)	0.16	0.19	0.83 (510)	.407	[-0.22, 0.54]

Testing-channel robustness. Campus-based and online sessions followed identical procedures. Re-estimating each focal outcome with testing channel (campus session / campus resident session / online network test) and its interaction with memory type showed no channel main effect (all $p \geq .29$) and no Memory Type × Channel interaction (all $p \geq .18$) in any study: Study 1 interaction $F = 1.55$, $p = .188$; Study 2 $F = 1.58$, $p = .181$; Study 3 $F = 0.76$, $p = .551$; Study 4 $F = 0.93$, $p = .449$; Study 5 $F = 0.96$, $p = .431$; Study 6 $F = 0.88$, $p = .475$.

Method note for the Study 5 dependent-correlation comparison (Table S11). The confidence interval for the difference between the two dependent correlations sharing MBF (data-sharing vs. transactional engagement) was obtained from a 5,000-resample percentile bootstrap with a fixed seed, resampling participants and recomputing both correlations and their difference in each resample (script: `bootstrap_indirect_effects.py`).

Standardized pairwise effect sizes (Cohen's d, pooled SD, 95% CIs) for focal outcomes.

Outcome	Comparison	d	95% CI
S1 trust	Relevant vs. No	0.40	[0.10, 0.70]
S1 trust	No vs. Intrusive	0.11	[-0.19, 0.40]
S1 trust	Relevant vs. Intrusive	0.51	[0.20, 0.81]

Outcome	Comparison	d	95% CI
S2 trust	Relevant vs. No	0.31	[0.03, 0.60]
S2 trust	No vs. Intrusive	0.58	[0.29, 0.87]
S2 trust	Relevant vs. Intrusive	0.89	[0.60, 1.19]
S4 trust (pooled)	Relevant vs. No	0.41	[0.20, 0.63]
S4 trust (pooled)	No vs. Intrusive	0.52	[0.30, 0.73]
S4 trust (pooled)	Relevant vs. Intrusive	0.92	[0.69, 1.14]
S5 data-sharing	Relevant vs. No	0.66	[0.39, 0.94]
S5 data-sharing	No vs. Intrusive	0.26	[-0.01, 0.53]
S5 data-sharing	Relevant vs. Intrusive	0.97	[0.68, 1.25]
S6 loyalty	Relevant vs. No	0.30	[0.04, 0.56]
S6 loyalty	No vs. Intrusive	0.28	[0.02, 0.54]
S6 loyalty	Relevant vs. Intrusive	0.59	[0.32, 0.85]
S6 opt-in	Relevant vs. No	0.33	[0.07, 0.59]
S6 opt-in	No vs. Intrusive	0.44	[0.18, 0.70]
S6 opt-in	Relevant vs. Intrusive	0.78	[0.51, 1.05]

Note. Uncorrected CIs; inferential decisions in the main text rest on the Tukey-adjusted comparisons (Table S8) and the Holm-adjusted focal tests above.

Assumption checks for the focal ANOVAs. Levene's tests assessed variance homogeneity across design cells; residual skewness, kurtosis, and Shapiro–Wilk tests assessed normality.

Study (focal outcome)	Levene W	p	Resid. skew	Resid. kurtosis	Shapiro–Wilk W	p
1 (trust)	0.65	.521	-0.11	-0.17	.991	.088
2 (trust)	0.12	.891	0.05	-0.04	.993	.210
3 (trust, 6 cells)	3.77	.002	0.16	-0.04	.995	.174
4 (trust, 6 cells)	1.09	.366	-0.13	0.35	.994	.056
5 (data-sharing)	1.68	.188	-0.11	0.04	.991	.046
6 (opt-in)	0.29	.745	-0.25	-0.25	.987	.004

Residual distributions were close to normal in every study (all $|\text{skew}| \leq 0.25$, all $|\text{kurtosis}| \leq 0.35$); the significant Shapiro–Wilk statistics in Studies 5 and 6 reflect trivial departures at large n. Variance homogeneity held in every study except Study 3, where the intrusive-memory/sleep-health cell had the largest spread ($SD = 1.34$). The Study 3 tests were therefore re-estimated with heteroscedasticity-robust (HC3) standard errors: the Memory Type × Scenario interaction remained significant (robust Wald $F = 4.02$, $p = .018$), as did the difference-in-differences contrast between the complete scenarios (estimate = 0.54, robust $SE = 0.26$, $z = 2.06$, $p = .039$, 95% CI [0.03, 1.06]). These tests establish a scenario-package difference, not an isolated effect of information sensitivity.

Supplementary Table S9b. Indirect-association models restricted to relevant versus intrusive memory (added in revision)

Because the no-memory condition presents no remembered content for participants to evaluate, its MBF score has no clear referent. We therefore re-estimated the Study 2 and Study 6 indirect-association models on the relevant-versus-intrusive subsample alone, in which every participant judged an actual memory cue. Memory type is coded 0 = intrusive, 1 = relevant. Confidence intervals are 95% percentile bootstrap intervals (5,000 resamples, fixed seed). Randomization supports the memory-type contrast, but the MBF-to-outcome and privacy-trust-to-outcome paths remain associational.

Study 2: memory type → MBF → trust ($n = 193$; 99 relevant, 94 intrusive).

Path	Estimate	SE	p
a (memory type → MBF)	1.612	0.150	$< .001$
b (MBF–trust association, controlling memory type)	0.443	0.063	$< .001$
c (total effect on trust)	0.903	0.146	$< .001$
c' (direct association with trust)	0.189	0.165	.254

Indirect association	Estimate	95% CI
Memory type → MBF → trust	0.714	[0.486, 0.964]

Study 6: memory type → MBF → privacy trust → loyalty / opt-in intention ($n = 227$; 115 relevant, 112 intrusive).

Path estimates: a_1 (memory type → MBF) = 1.615 ($p < .001$); d_{21} (MBF–privacy trust association) = 0.437 ($p < .001$); b_2 (privacy trust–outcome association) = 0.441 for loyalty and 0.480 for opt-in (both $p < .001$).

Outcome	Total effect c	Direct association c'	Serial indirect association & 95% CI
Loyalty intention	0.558 ($p < .001$)	0.074 ($p = .591$)	0.311 [0.203, 0.436]
Opt-in intention	0.749 ($p < .001$)	0.386 ($p = .007$)	0.339 [0.228, 0.473]

Note. On the subsample in which the intermediate appraisal has an unambiguous referent in every cell, the indirect associations are larger than the corresponding relative indirect associations estimated against the no-memory reference, and the direct association is not significant for trust (Study 2) or loyalty intention (Study 6). For opt-in intention a direct association remains. As in the main text, these are same-session associations: randomization licenses the causal interpretation of the memory-type contrast but not the ordering of MBF, privacy trust, or the outcomes. Accordingly, we do not interpret these decompositions as causal mediation.

Supplementary Note S14. Complete measurement documentation (added in revision)

All participant-facing materials were administered in Simplified Chinese. This note documents the thirteen constructs administered in the six experiments; the supplemental validation sample and its nine-item IUIPC measure are described separately in Table S5b. Throughout this note, the administered Chinese wording is given alongside the verified English back-translation; all other content is in English.

S14.1 Administration and scoring

Parameter	Specification
Language of administration	Simplified Chinese for all instructions, assistant messages, and questionnaire items
Response format	Seven-point agreement scale, presented as a one-item-per-row grid
Anchor labels	1 = 完全不同意 (strongly disagree), 7 = 完全同意 (strongly agree), midpoint 4 labelled 一般 (neutral)
Label coverage	Endpoints and midpoint labelled; scale points 2, 3, 5, and 6 displayed as numerals only
Item direction	All focal items positively keyed. Surveillance concern is the only construct keyed toward greater concern; no direction prompt preceded it and no reverse scoring was applied before averaging
Scoring rule	In the six experiments, each construct score is the unweighted mean of its three items; no imputation was performed, and cases missing a focal item were removed at screening. In the validation sample, MBF, warmth, surveillance concern, and trust are three-item means, whereas IUIPC is the mean of nine items
Block presentation order	Fixed and identical across the six studies: chat → MBF items (where measured) → trust items → purchase intention and continued use (Study 1) → platform-level judgments → downstream engagement and intention measures → demographics
Item order within a construct	Fixed, not randomized
Translation procedure	Independent forward translation into Simplified Chinese and back-translation into English by a second translator, with discrepancies reconciled by the author team (Brislin, 1970)
Record source	Administered wording recovered from the survey-platform project export (questionnaire builder, June 2026) and reconciled against the retained item-level response data in the de-identified analysis files

S14.2 Item inventory

All thirteen constructs administered in the six experiments have administered Chinese wording and a verified English back-translation. The wording for the constructs measured in Studies 1–4 and the

auxiliary measures was retained in the archived questionnaire; the wording for the six downstream scales introduced in Studies 5 and 6 was recovered from the original survey-platform project records (questionnaire export, June 2026) and reconciled against the retained item-level response data.

Construct	Studies	#	Administered Chinese wording	English back-translation
Memory Boundary Fit	1, 2, 4, 5, 6	1	助手记住的信息与本次交互是契合的。	The assistant’s remembered information fit the current interaction
		2	在这个情境下，使用这些被记住的信息是恰当的。	The remembered information was appropriate to use in this situation
		3	就这项任务而言，这种记忆的使用没有超出我可以接受的范围。	The memory use stayed within an acceptable boundary for this task
Trust	1, 2, 3, 4	1	我愿意信任这个助手。	I would trust this assistant
		2	这个助手看起来是可靠的。	This assistant seemed dependable
		3	我可以放心地依赖这个助手。	I would feel comfortable relying on this assistant
Purchase intention	1	1	我会考虑通过这个助手购买。	I would consider purchasing through this assistant
		2	我很可能会采纳它给出的推荐。	I would be likely to follow the recommendation
		3	这次交互提高了我的购买意愿。	The interaction increased my willingness to buy
Continued use	1	1	我会继续使用这个助手。	I would continue using this assistant
		2	遇到类似的决策，我还会再来使用它。	I would return to this assistant for similar decisions
		3	我会让这个助手保持启用状态。	I would keep this assistant enabled
Relational warmth	2	1	这个助手显得很用心。	The assistant seemed attentive
		2	这个助手显得很体贴。	The assistant seemed considerate
		3	这个助手看起来理解使用者。	The assistant seemed to understand the user
Surveillance concern	2	1	这个助手似乎知道得太多了。	The assistant seemed to know too much
		2	这次交互让我有被监视的感觉。	The interaction made me feel monitored
		3	这种记忆的使用让我产生了隐私方面的担忧。	The memory use raised privacy concerns
Privacy trust	6	1	我愿意信任该平台对我的信息的处理方式。	I would trust the platform’s handling of my information
		2	该平台在处理所存储的信息时是负责任的。	The platform seemed responsible with stored information

Construct	Studies	#	Administered Chinese wording	English back-translation
		3	该平台看起来尊重用户隐私。	The platform seemed respectful of privacy
Transactional engagement	5	1	我会考虑采纳这个助手推荐的购买方案。	I would consider following the purchase recommended by this assistant
		2	这次交互让我更愿意通过它完成购买。	This interaction made me more willing to complete the purchase through it
		3	我有打算按照它的推荐去下单。	I intend to place an order based on its recommendation
Data-sharing engagement	5	1	我愿意向这个助手提供更多我的偏好信息。	I would be willing to provide more of my preference information to this assistant
		2	为了得到更好的推荐，我同意它继续使用我之前的对话内容。	I agree to its continued use of my prior conversation content for better recommendations
		3	我不介意授权它访问更多与本次任务相关的数据。	I would not mind authorizing it to access more data relevant to this task
Relational engagement	5	1	我愿意继续和这个助手交流下去。	I would be willing to keep interacting with this assistant
		2	我愿意把它当作长期可信赖的购物伙伴。	I would treat it as a long-term, trustworthy shopping companion
		3	以后有需要我还想再来找它。	I would return to it when I have future needs
Loyalty intention	6	1	我打算持续使用这个助手的记忆功能。	I intend to keep using this assistant's memory feature
		2	我会把它推荐给身边有需要的人。	I would recommend it to people I know who might need it
		3	换到其他平台时，我也会希望有类似的记忆功能。	When using other platforms, I would want a similar memory feature
Opt-in intention	6	1	我愿意主动开启该助手记住我的偏好的功能。	I would actively opt in to letting the assistant remember my preferences
		2	我同意让它在未来的对话中调用我的历史信息。	I agree to let it retrieve my historical information in future conversations
		3	我希望它默认为我保留相关的交互记录。	I would like it to retain relevant interaction records for me by default
Platform trust	6	1	我对这个平台本身是信任的。	I trust this platform itself
		2	这个平台在保护用户信息方面做得让人放心。	This platform is reassuring in how it protects user information

Construct	Studies	#	Administered Chinese wording	English back-translation
		3	我认为这个平台是值得长期信赖的。	I consider this platform trustworthy over the long term

S14.3 Source of each adaptation

Construct	Adapted from	Nature of the adaptation
Trust; platform trust	McKnight et al. (2002)	Referent changed from a web vendor to the AI shopping assistant (trust) or the platform behind it (platform trust)
Purchase intention; transactional engagement	Pavlou (2003)	Referent changed to the assistant-mediated purchase in the focal scenario
Continued use; relational engagement; loyalty intention	Komiak and Benbasat (2006)	Referent changed from a personalized recommendation agent to the memory-enabled assistant
Privacy trust	Culnan and Armstrong (1999)	Procedural-fairness items reworded to address the platform's handling of stored conversational information
Surveillance concern; data-sharing engagement; opt-in intention	Malhotra et al. (2004), IUIPC	Collection and control subscales reworded from general online data practices to the assistant's retention and reuse of conversation content
Relational warmth	Komiak and Benbasat (2006)	Personalization and social-presence items reworded to describe the assistant's attentiveness in the focal exchange
Memory Boundary Fit	Newly written for this research, derived from contextual integrity (Nissenbaum, 2010)	Not adapted from an existing scale; validation reported in Table S5b

S14.4 Documentation status

All thirteen constructs administered in the six experiments have complete documentation of their administered Chinese wording, verified English back-translation (S14.2), source or theoretical basis (S14.3), response format and anchors (S14.1), and scoring rule. Item-level data availability differs across files, as recorded below; the validation sample is documented separately in Table S5b.

Documentation element	Status
Item-level response data	Retained for the experimental outcome measures and for all validation-sample measures; experimental MBF and some auxiliary appraisals are retained as composite scores only
Documented construct domain	Yes, for all measures
Administered Chinese wording	Yes, for all measures (S14.2)
Verified back-translation	Yes, for all measures (S14.2)
Source of adaptation	Yes, for all measures (S14.3)
Response format and anchors	Seven-point agreement scale, identical across the thirteen experimental measures (S14.1)
Scoring rule	Unweighted mean of three items for the thirteen experimental measures; the validation-sample IUIPC composite uses nine items
Reliability	Summarized in the main text where available; MBF validation evidence is in Table S5b and downstream-scale evidence in S14.5
Item-level structural evidence	Reported for the validation measures in Table S5b and for the six selected downstream scales in S14.5; no broader item-level structural claim is made for the other experimental measures

S14.5 Item-level psychometric evidence

A three-indicator one-factor model is just-identified, so the standardized loadings below follow in closed form from the inter-item correlations; composite reliability and average variance extracted are computed from those loadings, but global one-factor model fit cannot be tested. All values were computed from the item-level responses in the public data files with `revision_analyses.py` (fixed seed 20260722), which is available from the corresponding author on request together with the other analysis scripts listed in Table S4. Item-level psychometrics are reported here for the six downstream scales selected for added documentation in Studies 5 and 6. The earlier constructs and auxiliary measures are documented in S14.2, and reliability coefficients available from their retained data are summarized in the main text or Table S5b. This subsection does not claim to provide item-level structural analysis for every experimental measure; experimental MBF and some auxiliary appraisals are retained only as composites.

Study	Scale	Item	*M*	*SD*	Skew	Item-rest *r*	Loading
5	Transactional engagement	`Trans_Item1`	4.60	1.21	-0.01	.747	.833
5		`Trans_Item2`	4.63	1.15	0.04	.721	.793
5		`Trans_Item3`	4.67	1.18	0.03	.757	.848
5	Data-sharing engagement	`DataShare_Item1`	4.28	1.18	-0.19	.738	.817
5		`DataShare_Item2`	4.32	1.24	-0.13	.752	.839
5		`DataShare_Item3`	4.39	1.22	-0.14	.741	.822
5	Relational engagement	`Rel_Item1`	4.40	1.15	-0.21	.727	.791

Study	Scale	Item	*M*	*SD*	Skew	Item-rest *r*	Loading
5		`Rel_Item2`	4.50	1.16	-0.16	.771	.857
5		`Rel_Item3`	4.37	1.15	-0.05	.770	.856
6	Loyalty intention	`Loyalty_Item1`	4.34	1.11	-0.10	.687	.780
6		`Loyalty_Item2`	4.34	1.16	-0.05	.702	.806
6		`Loyalty_Item3`	4.29	1.11	0.10	.689	.783
6	Opt-in intention	`OptIn_Item1`	4.32	1.13	-0.10	.705	.771
6		`OptIn_Item2`	4.43	1.11	-0.22	.744	.829
6		`OptIn_Item3`	4.34	1.19	-0.23	.762	.863
6	Platform trust	`PlatformTrust_Item1`	3.76	1.15	0.09	.725	.825
6		`PlatformTrust_Item2`	3.71	1.20	-0.05	.680	.752
6		`PlatformTrust_Item3`	3.78	1.16	-0.05	.732	.836

Scale-level summary.

Study	Scale	α	CR	AVE	Loading range	Min item-rest *r*	Inter-item *r* range
5	Transactional engagement	.86	.86	.68	.79–.85	.72	.66–.71
5	Data-sharing engagement	.87	.87	.68	.82–.84	.74	.67–.69
5	Relational engagement	.87	.87	.70	.79–.86	.73	.68–.73
6	Loyalty intention	.83	.83	.62	.78–.81	.69	.61–.63
6	Opt-in intention	.86	.86	.68	.77–.86	.71	.64–.72
6	Platform trust	.85	.85	.65	.75–.84	.68	.62–.69

Composite correlations among the affected scales. Study 5 (N = 318): transactional–data-sharing $r = -.02$; transactional–relational $r = .03$; data-sharing–relational $r = .12$. Study 6 (N = 343): loyalty–opt-in $r = .32$; loyalty–platform trust $r = .13$; opt-in–platform trust $r = .24$; privacy trust with loyalty $r = .52$, with opt-in $r = .52$, with platform trust $r = .25$.

S14.6 Summary

All six downstream scales were internally consistent (α and CR $\geq .83$) and above the conventional AVE threshold of .50, with every loading $\geq .75$ and every corrected item-rest correlation $\geq .68$. These values are consistent with coherent three-item scoring representations, but the just-identified one-factor models do not provide a test of global unidimensional fit. Inter-item correlations fell between .61 and .73; none approached unity, and no single item carried the scale by itself. The near-zero correlation

between transactional and data-sharing engagement in Study 5 ($r = -.02$) further indicates that these were not interchangeable measures, which is a precondition for the H5 comparison rather than an artefact of it. Together with the administered wording in S14.2 and the source adaptations in S14.3, this evidence supports treating the six composites as internally coherent measures of distinguishable domains, while remaining appropriately limited by the three-item structure.

Supplementary Note S15. Incremental validity of MBF over concurrently measured appraisals (added in revision)

The reviewers asked whether MBF adds explanatory power beyond adjacent evaluative constructs. Two samples measured such constructs alongside MBF and trust and therefore permit a direct incremental-validity test: Study 2 ($N = 289$), which measured relational warmth and surveillance concern, and the supplemental MBF validation sample ($N = 234$), which measured IUIPC privacy concern, relational warmth, and surveillance concern. In each sample we regressed trust hierarchically, entering the adjacent appraisals first and MBF second. The archived analysis also contains reverse-order checks for warmth and surveillance concern in Study 2 and for surveillance concern in the validation sample; it does not report reverse-order increments for every validation-sample appraisal. Standardized coefficients are from the full model.

Sample	Step 1 predictors	Variable added	R^2 step 1	R^2 step 2	ΔR^2	F change (p)
Study 2 ($n = 289$)	Warmth, surveillance concern	MBF	.127	.326	**.199**	$F(1, 285) = 84.30$ ($p < .001$)
Study 2	MBF, surveillance concern	Warmth	.326	.326	.000	$F(1, 285) = 0.03$ ($p = .854$)
Study 2	MBF, warmth	Surveillance concern	.326	.326	.000	$F(1, 285) = 0.03$ ($p = .854$)
Validation ($n = 234$)	IUIPC, warmth, surveillance concern	MBF	.256	.456	**.200**	$F(1, 229) = 84.33$ ($p < .001$)
Validation	IUIPC, warmth, MBF	Surveillance concern	.455	.456	.001	$F(1, 229) = 0.54$ ($p = .465$)

Standardized coefficients in the full models: Study 2, $\beta_{\text{MBF}} = .56$ ($p < .001$), $\beta_{\text{warmth}} = .01$ ($p = .854$), $\beta_{\text{surveillance}} = -.01$ ($p = .854$); validation sample, $\beta_{\text{MBF}} = .55$ ($p < .001$), $\beta_{\text{warmth}} = .19$, $\beta_{\text{IUIPC}} = -.10$, $\beta_{\text{surveillance}} = .05$ ($p = .465$).

In both samples MBF explained approximately 20 percentage points of additional variance in trust beyond the adjacent appraisals entered at Step 1. In Study 2, the zero-order associations of warmth ($r = .25$) and surveillance concern ($r = -.32$) with trust were absorbed after MBF entered, and the reverse-order increments for both were below .001 ($p = .854$). In the validation sample, the reported reverse-order increment for surveillance concern was .001 ($p = .465$); corresponding reverse-order increments were not reported for warmth or IUIPC. The primary incremental results support the limited conclusion that MBF contributed information beyond the measured appraisal set in each sample. They do not establish causal priority or address the unmeasured alternatives.

What this test does and does not establish. It establishes incremental validity over the adjacent appraisals that were measured concurrently: general privacy concern, relational warmth, and

surveillance concern. It does not address perceived usefulness, creepiness, intrusiveness, information relevance, or perceived norm violation, none of which were measured in these studies. Because the analyses are cross-sectional and MBF and trust were reported in the same session, the incremental variance is also subject to common-method variance. The test therefore narrows, but does not close, the discriminant-validity question, and the design of the study needed to close it is specified in Note S16.

Supplementary Note S16. Systematic conceptual comparison of MBF with its nearest neighbours (added in revision)

MBF is an interaction-specific judgment of whether a particular piece of remembered content belongs in the current exchange. The table below compares it with the constructs that offer the most plausible rival accounts of the same reaction, on the dimensions that distinguish them: the object being judged, the level at which the construct operates, its affective character, and the observable prediction that separates it from MBF. The final column states the measurement that would adjudicate between the two.

Construct	Object of judgment	Level	Where it diverges from MBF	Discriminating test
Memory Boundary Fit	Whether this remembered content belongs in this exchange	Interaction-specific	—	—
Perceived usefulness	Whether the content helps the current decision	Interaction-specific	Content can be useful yet out of place (a correct inference from sensitive data), or harmless yet useless	Cross the two orthogonally: a useful-but-misplaced cue should lower MBF while raising usefulness
Information relevance	Topical match between content and task	Interaction-specific	Relevance is a property of the content; fit additionally depends on how the assistant came to know it	Hold topical relevance constant and vary the information's source (in-context disclosure vs. cross-context inference)
Perceived intrusiveness	Interruption of, or forced exposure during, an ongoing task (Edwards et al., 2002)	Interaction-specific	Intrusiveness concerns the interruption; MBF concerns the content's entitlement to appear at all	Deliver an equally interruptive but contextually appropriate cue; intrusiveness should rise without MBF falling

Construct	Object of judgment	Level	Where it diverges from MBF	Discriminating test
Creepiness	Unsettled feeling at a lawful but norm-ambiguous practice (Tene & Polonetsky, 2014; Petrova et al., 2026)	Diffuse affective	Creepiness is an affective reaction; MBF is an appropriateness judgment that may be rendered without affect	Measure both across separated occasions and compare plausible temporal orderings
Perceived norm violation	Whether a rule governing the information has been broken (Petronio, 2002, 2013)	Interaction-specific	Norm violation presumes an identifiable rule and a co-owner; MBF can register misfit without any rule being nameable	Use a cue that fits no established norm in either direction; MBF should still discriminate
Expectancy violation	Departure from what the partner was expected to do (Burgoon, 1993)	Interaction-specific	Violations can be positive; an unexpectedly apt memory violates expectancy while raising MBF	Include a positively valenced surprise cue; the two constructs should dissociate in sign
Surveillance concern	Belief that one is being monitored	Interaction-specific	Concerns the monitoring apparatus, not the appropriateness of a single reuse	Measured in Study 2; MBF absorbed its association with trust (Note S15)
General privacy concern	Disposition toward data collection and use (Smith et al., 1996; Malhotra et al., 2004)	Dispositional	Stable across exchanges; MBF varies within a single conversation	Measured in the validation sample; MBF retained incremental validity over IUIPC (Note S15)

Status of the comparison. The bottom two rows are supported empirically in the present data (Note S15 and Table S5). The remaining rows are conceptual: they specify how MBF is intended to differ and what evidence would falsify the distinction, but they are not tested here. The decisive study would measure MBF alongside perceived usefulness, creepiness, intrusiveness, information relevance, and perceived norm violation in a single sample, establish a factor structure across all six appraisals, and test whether MBF retains incremental validity in its association with trust. Until that study is run, MBF's distinctiveness from these unmeasured appraisals remains a conceptual claim rather than an established psychological mechanism.
